# Ultrafast bridge planarization in donor-π-acceptor copolymers drives intramolecular charge transfer

**DOI:** 10.1038/s41467-017-01928-z

**Published:** 2017-11-23

**Authors:** Palas Roy, Ajay Jha, Vineeth B. Yasarapudi, Thulasi Ram, Boregowda Puttaraju, Satish Patil, Jyotishman Dasgupta

**Affiliations:** 1 Department of Chemical Sciences, Tata Institute of Fundamental Research, Mumbai, 400005 India; 2 Department of Nuclear and Atomic Physics, Tata Institute of Fundamental Research, Mumbai, 400005 India; 3 Solid State and Structural Chemistry Unit, Indian Institute of Science, Bangalore, 560012 India

## Abstract

Donor-π-acceptor conjugated polymers form the material basis for high power conversion efficiencies in organic solar cells. Large dipole moment change upon photoexcitation via intramolecular charge transfer in donor-π-acceptor backbone is conjectured to facilitate efficient charge-carrier generation. However, the primary structural changes that drive ultrafast charge transfer step have remained elusive thereby limiting a rational structure-function correlation for such copolymers. Here we use structure-sensitive femtosecond stimulated Raman spectroscopy to demonstrate that π-bridge torsion forms the primary reaction coordinate for intramolecular charge transfer in donor-π-acceptor copolymers. Resonance-selective Raman snapshots of exciton relaxation reveal rich vibrational dynamics of the bridge modes associated with backbone planarization within 400 fs, leading to hot intramolecular charge transfer state formation while subsequent cooling dynamics of backbone-centric modes probe the charge transfer relaxation. Our work establishes a phenomenological gating role of bridge torsions in determining the fundamental timescale and energy of photogenerated carriers, and therefore opens up dynamics-based guidelines for fabricating energy-efficient organic photovoltaics.

## Abstract (web summary)

Tracking and understanding charge transfer process is central yet challenging to designing efficient organic photovoltaics. Roy et al. monitor real-time structural changes in donor-π-acceptor polymer backbone, and enumerate the role of π-bridge torsions during intramolecular charge transfer.

## Introduction

Organic photovoltaics (OPVs) that comprise of conjugated polymers both as light absorbers and its charge transport layer promises to be an economically viable alternative to the Si-based solar cells^1–3^. In recent years, facile chemical synthesis of low bandgap donor−acceptor copolymers has resulted in extending the window for solar light absorption in OPVs through hybridization of donor-type and acceptor-type molecular orbitals^4–6^. In spite of these economically viable materials, the desired increase in the power conversion efficiency beyond 11% has been difficult to achieve^7^. The major issue plaguing the device efficiency is the loss of photon energy during the early events of photoinduced exciton-to-charge conversion. The most wide-absorbing OPV with bandgap of 1.5 eV displays a maximal open circuit voltage (*V*_oc_) of 0.79 V thereby resulting in 47% loss of photon energy^8^. Due to the large intrinsic exciton binding energy (above 200 meV) in such organic materials, energy stabilized charge transfer (CT) excitonic states are employed to separate the electrons and holes at inevitably a lower potential. If however the CT reaction is tailored to be temporally faster than all energy relaxation processes encountered by the nascent exciton, energy loss pathways could be minimized. Therefore synthetic control on ultrafast generation of high energy or hot CT states together with optimization of the charge injection pathways at electrode interfaces would lead to high device *V*_oc_^9^.

**Fig. 1:**
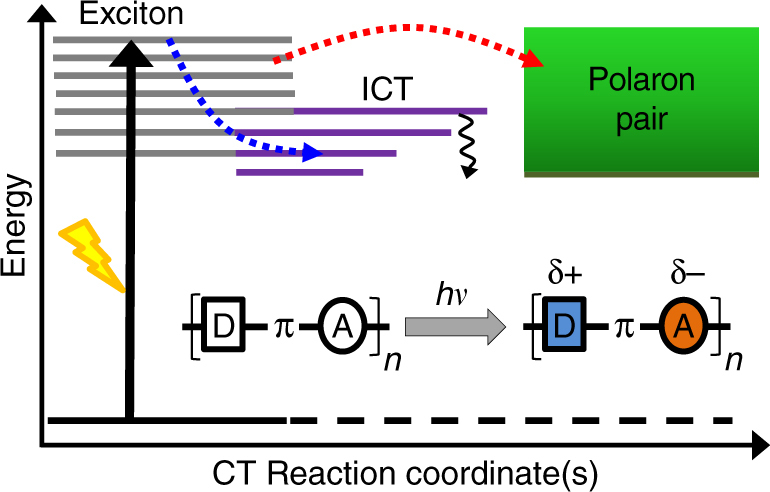
Energetics of photoinduced processes in neat low-bandgap donor-π-acceptor copolymers. Photogenerated excitons can lead to ultrafast intramolecular charge transfer (ICT) and polaron pair states

The material design community has encountered a tremendous challenge in optimizing the charge generation efficiency in OPVs without having a well-defined structure−activity correlation for contemporary donor−acceptor copolymers. The major issue that limits such a correlation is the lack of proper structural understanding of the exciton dynamics and the resulting formation of charge-pair states. Therefore, it is imperative to understand the fundamental limits of CT timescales and the energy associated with these CT states in the context of a given donor−acceptor backbone by tracking exciton dynamics. With the advent of ultrafast laser technology, it has been possible to optically probe the dynamics of photogenerated excitons in conjugated organic polymers with femtosecond time-resolution and high spatial accuracy^10, 11^. Recent work from various groups have suggested that in donor-π-acceptor copolymers where the donor and acceptor moieties are separated by the π-bridge, intramolecular charge transfer (ICT) character in the excited state with large dipole moment promotes the formation of polaron pairs, the precursor state for charge carriers^12, 13^. It has also been shown that spatial separation of the donor and acceptor groups by intervening bridge prolongs the recombination time of the charge pairs^12^. However, increasing the backbone degrees of freedom can reduce the effective energy of the charge carriers simply due to multiple relaxation pathways and deep CT stabilization. Therefore, optimizing the complex interplay between ultrafast polaron pair generation and exciton relaxation pathways (see Fig. 1) via elucidation of the CT reaction coordinate (RC) is the key in optimizing performance of organic photovoltaic devices^14^.

Multiple studies have indicated that the instantaneously generated hot excitons in bulk heterojunction solar cells have large delocalization lengths which lead to coherent generation of charges at polymer:fullerene interfaces^15–21^. These photogenerated hot excitons on pristine homopolymer backbone can go through various temporally segregated relaxation pathways: exciton self-trapping in 30−100 fs, local torsional relaxation within 200−500 fs, and excitonic energy transfer in one to few tens of picoseconds^22–29^. Low-bandgap polymers have been however difficult to probe due to the complexity of polymer excited states having varying degrees of CT character. Banerji et al.^30^ who provided the first report on low bandgap polymer PCDTBT (poly[N-11′′-henicosanyl-2,7-carbazole-alt-5,5-(4′,7′-di-thienyl-2′.1′.3′-benzothiadiazole)]) demonstrated about 200 fs Stokes shift in emission concomitant with loss in anisotropy suggesting an ultrafast relaxation mechanism in the donor-π-acceptor backbone. Greg Scholes and co-workers performed two-dimensional electronic spectroscopy (2D-ES) on PCDTBT and observed an increase in the dipole moment which was attributed to the localization of the ICT state within 200 fs timescale^14, 26^. Wavelength selective transient absorption (TA) carried out by Fazzi et al. showed that ultrafast electronic relaxation through conical intersection can also lead to relaxation in the excitonic manifolds^31^. However, the large inhomogeneity in the spectral features of the polymer excited states prevents unambiguous assignment of multiple types of CT states and their dynamics. Additionally, the role of the structural elements in the donor-π-acceptor backbone has not been addressed directly thus precluding any information about the ICT RCs.

**Fig. 2:**
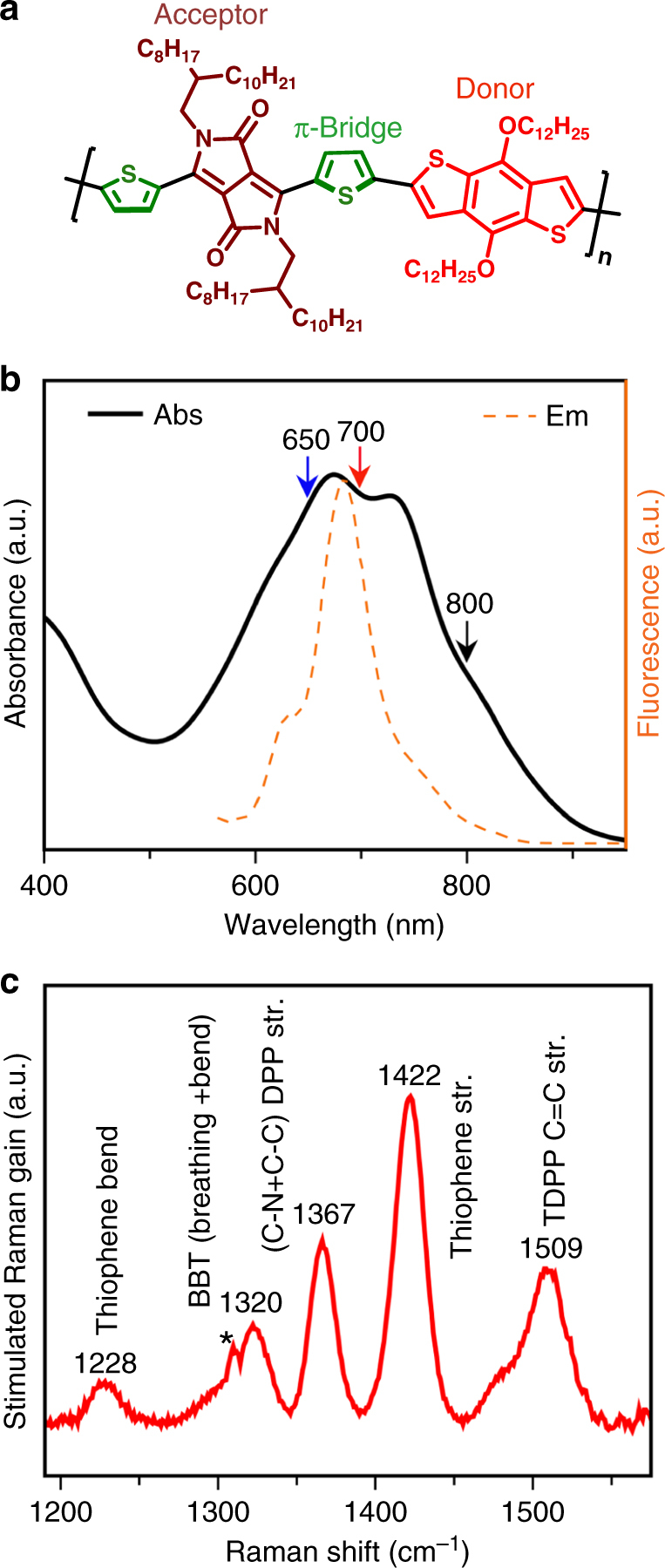
Spectroscopic characterization of TDPP-BBT copolymer. **a** Structural representation of TDPP-BBT copolymer. **b** Steady-state absorption of the 90 μm copolymer solution in chlorobenzene is represented by black curve while the emission spectra is represented by orange dotted curve where 570 nm is the excitation wavelength; 650, 700, and 800 nm are the pump wavelength used for transient absorption measurements. **c** Ground state stimulated Raman spectra of TDPP-BBT polymer in chlorobenzene where 816 nm is the excitation pulse. Asterisk mark represents instrumental artifact

 Femtosecond stimulated Raman spectroscopy (FSRS)^32^, a structure-sensitive vibrational technique, provides both good spectral (10 cm^−1^) as well as high temporal resolution (about 50 fs) for tracking photogenerated excitons and charges in the polymer backbone as demonstrated by Bragg and co-workers^33–36^. Recently, Sophia Hayes and co-workers reported transient Raman signatures of donor-π-acceptor copolymer in their exciton and polaronic states^37^. They observed 1–3 ps evolution of the exciton to a partial charge transfer state within the donor and acceptor units which however is much slower than the expected torsional dynamics of the backbone^14, 26^. In order to observe the ultrafast generation of the ICT exciton and its structural imprints in the transient Raman measurements, here we chose diketopyrrolopyrrole (DPP)-based low bandgap polymer with a defined molecular π-bridge in between the donor and acceptor units as shown in Fig. 2a. DPP-based polymers are commonly used in organic electronics because of large absorption cross-section and its widely tunable range along with good charge carrier mobilities^38, 39^. Bulk heterojunction solar cells made out of TDPP-based copolymers have reached devices efficiencies of around 9.4% which highlights the potency of the DPP-based molecular architectures^40^. The thiophene unit in the TDPP part acts as a structural π-bridge to electronically couple the donor benzobithiophene (BBT) and the acceptor DPP units (see Fig. 2a). Here we selectively probe the transient structural changes of poly(thiophene-diketopyrrolopyrrole-benzobithiophene) (TDPP-BBT) polymer backbone during its photoexcited evolution to the ICT state using resonance-enhanced FSRS. Our results suggest a sub-picosecond exciton relaxation driven by the torsional motion of the thiophene bridge, a chemical moiety omnipresent in majority of DPP-based polymers^38, 41^, establishes a delocalized ICT character to the backbone centric excitation.

## Results

### Steady-state electronic and vibrational Raman spectroscopy

The structure of TDPP-BBT copolymer that we have used is shown in Fig. 2a. TDPP-BBT class of low bandgap polymers has typically an absorption range in the visible due to well-coupled acceptor DPP and donor BBT^42^. The large molar extinction coefficient along with primarily visible absorption of the TDPP unit (see Supplementary Fig. 22 for only TDPP monomer absorption) dominates the absorption spectrum of the TDPP-BBT polymeric system. The thiophene unit which is an intrinsic part of the TDPP unit serves as a structural bridge between DPP and BBT thereby avoiding any steric repulsion between the side-chains on the individual donor and acceptor units^12, 38, 43^. It also enables long-range charge separation between the donor and acceptor so as to ensure long-lived charge transfer states as has been reported in other donor-π-acceptor copolymers^12^.

Figure 2b shows the broad steady-state absorption spectra of TDPP-BBT copolymer in chlorobenzene. The characteristic π−π* transition of the polymer shows a maxima at around 670 nm. Concentration-dependent measurements performed from 2 to 90 μm did not show any significant change in the absorption spectra (Supplementary Fig. 10). Steady-state fluorescence measurements under dilute conditions reveal a narrow emission band (as shown in Fig. 2b) which appears to be overlapping with the red-part of the absorption feature. Such kind of mid-bandgap emission is typical in many donor−acceptor copolymers^42, 44, 45^, and have been explained by ground state heterogeneity in the polymeric solution^44^. The excitation spectrum (Supplementary Fig. 11) showed that the blue part of the absorption spectrum primarily constitutes the emissive population while the red-absorbing population has quenched emission. In addition, it has been shown previously that TDPP-BBT in solution shows distinct solvatochromic shift in its electronic absorption and emission spectra owing to a prominent ICT character in the excited state^42^.

Figure 2c describes ground state stimulated Raman (GSR) spectrum of the polymer in chlorobenzene solution collected with 816 nm Raman pump excitation. The spectrum shows five distinct features in between 1200 and 1600 cm^−1^ spectral detection window after subtraction of the solvent modes (see Supplementary Fig. 1 for GSR data processing). The stimulated Raman detection helps in increasing the signal-to-noise ratio of the Raman spectrum when compared to the spontaneous Raman measurements performed with 532 nm CW laser excitation which show substantial emission background (see Supplementary Fig. 12). Assignment of the modes were done by computing the vibrational frequencies of the ground state optimized structure by density functional theory (DFT) (see Supplementary Figs. 8–9) and corroborated with the reported values obtained from the Raman database on thiophene oligomers and polymers^42, 46–49^. The 1228 cm^−1^ mode has main contribution from the C−H bending coupled to ring stretches while the 1422 cm^−1^ mode corresponds to C=C stretching of thiophene ring. The 1320, 1367, and 1509 cm^−1^ modes correspond to the BBT C=C stretching admixed with ring breathing/bending, DPP C-C/C-N stretching, and TDPP C=C stretching, respectively. These chromophore-specific modes present an excellent opportunity to probe the torsional dynamics of the donor-π-acceptor backbone in the excited state. The intensities of the ground state modes indicate the resonance Raman cross-section which partially signifies the excited state slope in the Franck−Condon region of the excited state potential surface^50^. To understand the electronic states and frequency changes in these modes upon photoexcitation, we have carried out wavelength selective TA and time-resolved FSRS measurements as described below.

### Transient absorption of TDPP-BBT solution

**Fig. 3:**
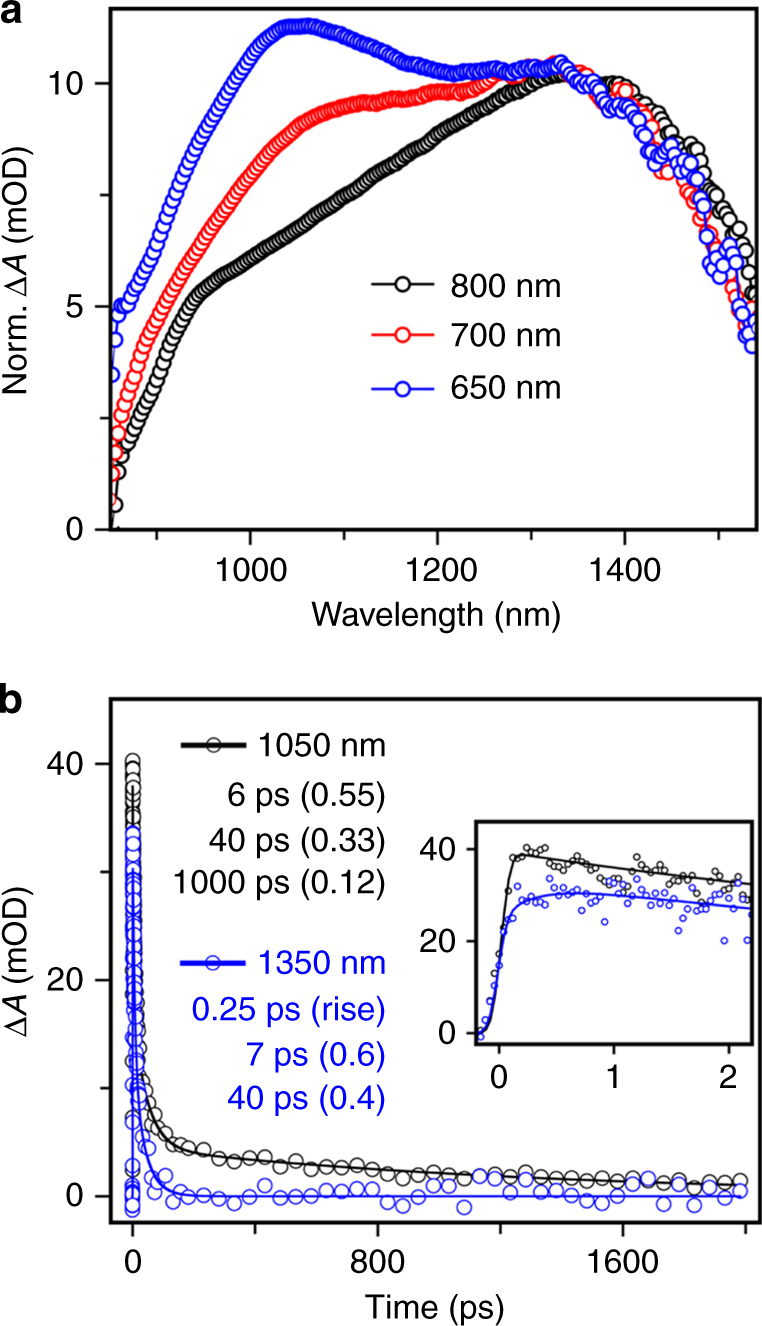
Transient absorption of TDPP-BBT in chlorobenzene. **a** Actinic pump wavelength-dependent (650, 700, and 800 nm) excited state absorption spectra at 1 ps; **b** kinetics fitting at 1050 nm and 1350 nm for 650 nm excitation showing the presence of a short-lived and long-lived species

The intrinsic linewidth obtained in the steady-state absorption and emission spectra complicates the understanding of the conformational heterogeneity present in the solution. In order to characterize the nature of polymer conformers present under the absorption envelope, we performed broadband near-IR femtosecond TA spectroscopy with three excitation wavelengths namely at 650, 700, and 800 nm (indicated by arrows in Fig. 2b). Using 650 nm femtosecond pulse excitation, TA spectra were recorded with an instrument response function (IRF) of about 90 fs at various pump-probe delays in the NIR region from 850 to 1550 nm. Photoexciting the pristine polymer solution in chlorobenzene results in a broad excited state absorption with two features at around 1050 and 1350 nm as shown in the blue trace of Fig. 3a. Both the features decay with different time-constants as indicated by the ∆*A* vs time plotted at the two peak positions in Fig. 3b (also see Supplementary Fig. 14). Kinetic fitting with a three exponential model showed that 1050 nm feature lives upto 1 ns while feature at 1350 nm has a shorter lifetime of about 40 ps. In order to unravel the nature of these states, we carried out time-correlated single photon counting (TCSPC) measurements of the polymer in chlorobenzene using the 630 nm excitation while collecting the emission at 690 nm. Our TCSPC data (see Supplementary Fig. 13) shows that there is long-lived singlet exciton with a mean lifetime of about 850 ps. Therefore, the broad blue absorbing feature at 1050 nm in the TA measurements can be assigned to singlet exciton of the polymer.

Excitation wavelength-dependent TA carried out at 700 and 800 nm which covers the red-region of the absorption spectrum showed an extremely important trend in the excited state absorption features. As shown in Fig. 3a, we compare the normalized ∆*A* spectrum at 1 ps obtained using 650, 700 and 800 nm pump respectively. The ratio of the 1050 to 1350 nm ESA features progressively decreases as the pump excitation is shifted more to the red-absorbing side of the absorption envelope. This clearly indicates that conformational heterogeneity leads to altered excited state dynamics with the generation of short-lived electronic excited states. The decay kinetics observed for both the 1050 and 1350 nm feature remains unaltered. A rise time of 150–250 fs is observed for the 1350 nm feature (see Supplementary Fig. 23). The ultrafast rise time can be explained by invoking a red-absorbing population of the polymers which have a fast non-radiative transition leading to a state with short lifetime. It has been reported previously that inter-strand interactions via aggregation leads to enhanced coupling with low lying charge transfer or polaronic states^51^. The presence of such polaron pair states was confirmed by comparing the absorption spectrum of the localized polymer hole polaron with the observed TA at 1350 nm. We find that the FeCl_3_ doping leads to formation of a localized hole polaron in the polymer with absorption spectrum (see Supplementary Fig. 15) that matches well with the red-absorbing TA feature. Although we do not spectrally observe the electron polaron, we cannot rule out its presence since it may be coulombically bound to the hole polaron.

To confirm further that the polaron pair state in the TA spectra arises due to inter-strand packing, we carried out concentration-dependent TA measurements. The 1 ps spectrum compared for polymer concentrations of 2, 6, and 90 μm polymer shows that the 1350 nm feature emerges at higher concentration of the polymer (see Supplementary Fig. 16). This allows us to clearly distinguish that optimized packing or inter-strand coupling at high concentration leads to polaronic state formation. Our results here on TDPP-BBT donor-π-acceptor copolymers are analogous to that obtained for PTB7 polymer by Faulvell et al. which also demonstrated mid-bandgap emission and conformational heterogeneity with short-lived self-aggregated excitonic states on the red-part of the absorption spectrum^26, 44^. Recently Bragg and co-workers have also reported heterogeneity arising from exciton localization on different spatial regions of the monomer strand^52^. We cannot exclude such a possibility in our case although our concentration dependence studies and chemical doping experiments support photoinduced polaronic states being formed. Due to strong spectral overlap between the exciton feature at 1050 nm and the polaronic transitions in 1350 nm in the TA data, it is difficult to ascertain the timescale of the relaxation processes through only pump-probe spectroscopy. To get precise molecular level information selectively of the exciton, we therefore performed time-resolved stimulated Raman measurements which would allow to selectively probe the singlet exciton population via resonance enhancement.

### FSRS of TDPP-BBT polymer

**Fig. 4:**
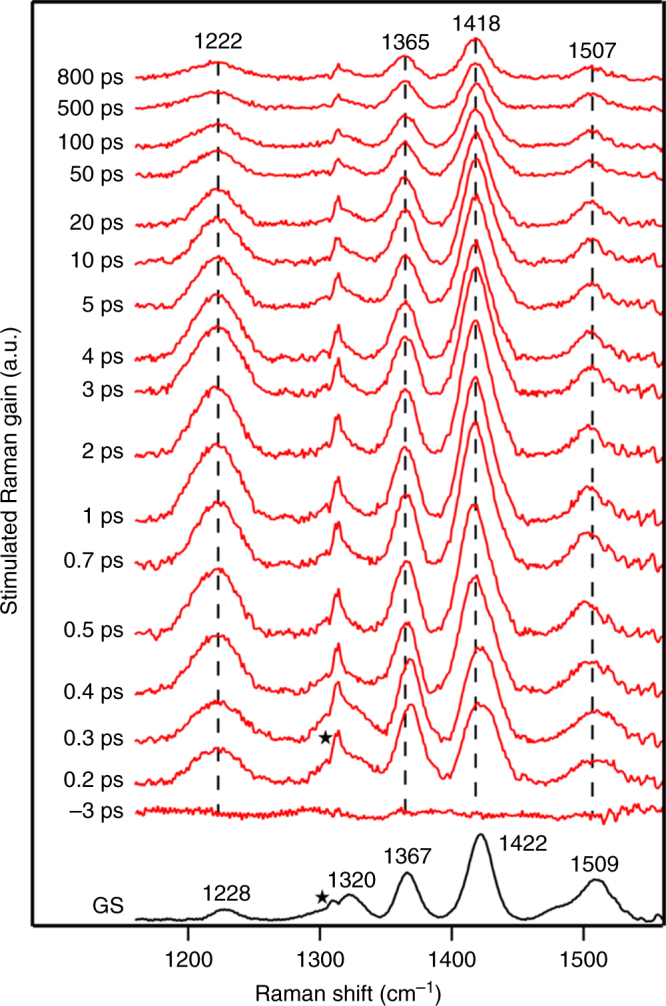
Femtosecond stimulated Raman spectra of TDPP-BBT in chlorobenzene. Excited state Raman spectra at different pump-probe delay times has been shown. The ground state and excited state Raman signals are represented by black and red traces respectively. Asterisk mark represents instrumental artifact

The structural evolution of polymer subsequent to the photoexcitation and the generation of the ICT character in the singlet manifold of states is probed by recording time-resolved Raman snapshots using FSRS. In order to be in-resonance selectively with the excitonic state, we collected Stokes shifted Raman data with 816 nm Raman pump pulse and compressed broadband probe pulse with a spectral range of 850−1300 nm. The excited state was populated using 0.25 mW actinic pulse centered at 650 nm to excite primarily the emissive population of the TDPP-BBT polymer in chlorobenzene. We plot the excited state Raman spectrum at various time delays in Fig. 4. The spectra represent the vibrational snapshots of only the excited population after removal of the ground state bleach fraction (details of FSRS data analysis is discussed in Supplementary Notes 4–5). The metric for recovering the pure excited state spectrum by adding back the ground state fraction at all the time-delays was constrained by the bleach recovery dynamics observed in the TA (see Supplementary Fig. 4). In fact, in the FSRS measurements, we find that the excited state species is long-lived till 800 ps indicating the singlet exciton species. The ground state Raman spectrum (Fig. 4, bottom trace in black) is plotted to compare the magnitude of the changes in vibrational frequencies and intensities of the modes. We observe the same number of excited state normal modes mirroring the ground state Raman spectrum. Both the peak area kinetics and ground state bleach recovery dynamics show long-lived state (see Supplementary Figs. 18–19) up to 800 ps indicating that the singlet exciton Raman modes have been resonantly enhanced. In order to support our assignment, we recorded the stimulated Raman spectrum of the hole-doped polymer after reaction with oxidant FeCl_3_. Supplementary Fig. 21 provides a comparative plot of the hole polaron Raman spectrum with the exciton spectra at 1 ps. It is evident that although the number of normal modes is the same, the peak positions are shifted with respect to that of the exciton. In addition, the ground state bleach recovery kinetics (shown in Supplementary Fig. 19) obtained while analyzing the FSRS data also shows a long-lived excited state that is in good agreement with the data obtained via TA. We do not see any evidence for decay of the excited state population which matches the polaron pair population decay dynamics obtained from TA. It should be mentioned that the 816 nm Raman pump does depopulate the polaron states through charge recombination back to the ground state (see Supplementary Figs. 17 and 35) without altering the lifetime of the exciton as confirmed by TA with Raman pump on. The Raman-induced depopulation supports our assignment that indeed the red-edge of the absorption spectrum (greater than 750 nm) of TDPP-BBT in solution is primarily from stacked configurations of the polymer which can generate polarons as primary excitations. Therefore, the recorded Raman spectra demonstrate the structural evolution of the polymer backbone in the singlet exciton state.

**Fig. 5:**
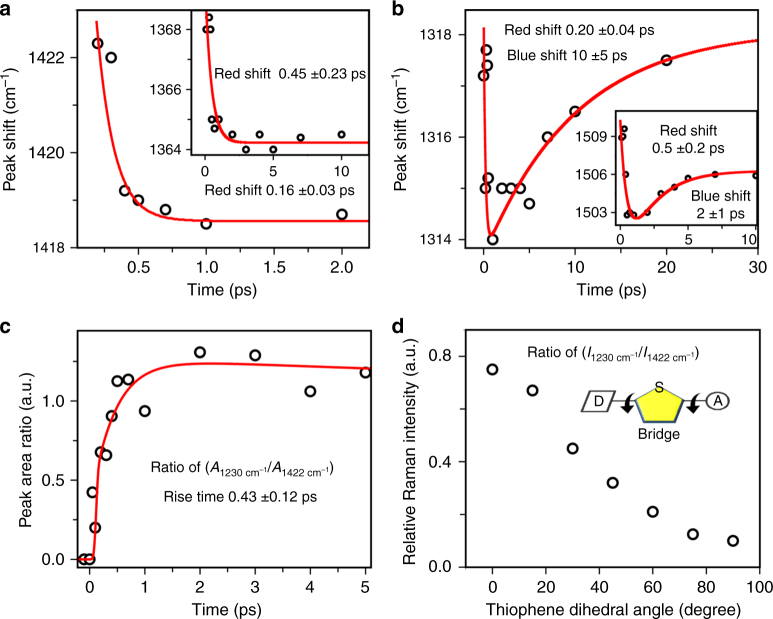
Transient changes in the Raman peak frequency and area. **a** Peak shift of 1422 and 1367 cm^−1^ mode (Inset) in about 160 and 450 fs timescale respectively; both the modes reflect red shifted spectra. **b** Peak shift of 1315 and 1503 cm^−1^ mode (inset). The 1315 cm^−1^ mode is red shifted in 200 fs and then blue shifted in 10 ps while 1503 cm^−1^ mode shows blue shift in 2 ps. **c** Relative peak area changes of 1228 cm^−1^ with respect to 1422 cm^−1^ mode occur in 430 fs timescale. **d** Relative Raman intensity change of 1228 cm^−1^ as a function of thiophene dihedral angle as obtained from DFT calculation

The ground state SRS spectrum showed thiophene centric modes at 1422 and 1228 cm^−1^ respectively. The mode at 1228 cm^−1^ arising from C−H bending coupled to ring stretches shows low Raman intensity in the ground state as compared to the C=C stretch mode at 1422 cm^−1^. After photoexcitation, both the modes show red-shift while the intensity ratio of the bend to the C=C stretch changed drastically. In addition, the bending mode becomes much broader with an increase in the Raman cross-section compared to the stretching mode. This can possibly be explained by the change in the dephasing timescale of the bending mode in the excited state via coupling to low frequency reactive modes like torsions. The C=C stretch mode evidently does show a 4 cm^−1^ shift within 500 fs while the peak has broader linewidth as compared to its ground state feature. The red-shifting occurs with a lifetime of about 160 fs (shown in Fig. 5a) indicating a structural change in the backbone which also results in Raman cross-section enhancement of the bending mode. The modest shifts obtained in our case are analogous to that reported by Bragg and co-workers for poly-3-cyclohexyl-1,4-methylthiophene polymer which also showed modest shifts of 5 cm^−1^ within 200 fs^36^. Additionally they reported that both the C=C stretch intensity in oligothiophenes does increase with increase in the backbone conjugation length^34^.

Interestingly the 1367 cm^−1^ mode that scores for the changes in electronic structure of the DPP ring also shows a red-shift with about 450 fs time constant (insets of Fig. 5a, b) possibly driven by structural change in the backbone. The imprint of the structural evolution is also sensed by the BBT moiety since the vibrational feature at 1320 cm^−1^ shows first a red-shift with 200 fs time-constant and a blue-shift with about 10 ps lifetime (see Fig. 5b; also see Supplementary Figs. 24–25 for peak fitting). The ultrafast frequency shift arises as a result of the structural evolution in the backbone while the picosecond blue-shift is assigned to vibrational cooling dynamics. The DPP C=C stretch mode at 1509 cm^−1^ however showed about 2 ps blue-shift indicating a rate dispersion in cooling timescales in the respective donor and acceptor moieties (Fig. 5b inset). Overall the FSRS spectra of the exciton present rich molecular-level information on the structural changes in the D-π-A backbone of the polymer leading to ICT and the localization of the ICT state within 1–10 ps.

Apart from the frequency shifts, the ratio of the integrated areas of the 1228 and the 1422 cm^−1^ feature shows 430 fs rise (see Fig. 5c) in the Raman intensities. We also observed peak area rise albeit less pronounced for DPP modes at 1367 and 1509 cm^−1^ with a 300–500 fs time-constant as shown in Supplementary Fig. 20. To get molecular insight into the structural transition, we have performed electronic structure calculations and computationally calculated the vibrational spectra of the model monomer in its ground state. The optimized geometry of the monomer at the B3LYP/6-31+ G level is obtained using Gaussian09 program. The optimized structure showed a 15 degree twist in the torsional angles between bridge thiophene and DPP rings as well as a similar number between the thiophene and BBT ring (see Supplementary Fig. 7 for optimized structure of TDPP-BBT). We found a satisfactory agreement in the frequency positions of the ground state Raman modes with the computationally extracted values as shown in Supplementary Figs. 8–9. In order to understand the change in Raman cross-section of the thiophene C−H bending mode we rotated the thiophene dihedral with respect to the DPP-BBT backbone and performed DFT calculation (B3LYP/6-31+ G) to determine the Raman activity changes. It is evident that the relative Raman activity of 1228 cm^−1^ mode with respect to the 1422 cm^−1^ mode increases with decreasing torsional angle as shown in Fig. 5c. Since the conjugation in the excited state has increased as evident from frequency shifts, the enhanced Raman activity of 1228 cm^−1^ relative to the C=C stretch in the excited FSRS spectra therefore is directly assigned to the planarization of donor-π-acceptor backbone via rotation of the thiophene structural bridge. Here it is important to acknowledge that there is no direct way of correlating excited state geometries with ground state torsional twists although fundamentally the Raman polarizability is directly correlated to the electron−phonon coupling strengths. It has been shown previously by Bragg and co-workers through very systematic experimental Raman and theoretical modeling on oligothiophenes that the Raman intensity increases with increasing conjugation in the ground state^53^. Irrespective of the potential energy surface on which the Raman intensities are calculated, increasing the conjugation length will lead to increased Raman polarizability due to the larger lengthscale of π-electron delocalization.

In order to appreciate the significance of observed backbone structural dynamics in dictating the photon-conversion efficiencies within devices, we carried out excited state FSRS measurements on spin-casted TDPP-BBT polymer film. The spin-casted films showed moderately red-shifted absorption with slight broadening as compared to the solution state spectrum (See Supplementary Fig. 26). Photo-excitation of the film was carried out in inert atmosphere of nitrogen and with low excitation fluence in order to avoid photodamage. Femtosecond TA spectra showed analogous excited state features as seen in solution with the exciton population peaking at 1040 nm while the polaron-pair ESA was centered at 1400 nm (see Supplementary Fig. 28). The exciton population decayed faster in the film which was also supported by shorter emission lifetime of about 400 ps in film obtained through TCSPC measurements (see Supplementary Fig. 27). The polaron-pair signatures dominate the transient spectra which is expected since a larger fraction of inter-chain packed polymers exist in the films. The FSRS measurements with 816 nm Raman pump on the films resonantly enhances the Raman signatures of the singlet exciton analogous to that in solution. The stimulated Raman spectra plotted in Supplementary Fig. 30 shows that all the ground state modes are red-shifted in films compared to solution. The altered Raman frequencies in film indicate enhanced conjugation due to solid state packing well correlated to the absorption spectrum. The FSRS spectra at various time-delays shown in Supplementary Fig. 30 demonstrate the similarity of the exciton dynamics both in solutions and films. The excitonic population decays within 500 ps, obtained from the integrated peak areas in FSRS measurements (see Supplementary Fig. 31), match well with the TCSPC measurements thereby conclusively identifying as the exciton state. The C=C stretching mode of the thiophene bridge which appears at 1414 cm^−1^ in the films gets dynamically red-shifted to 1408 cm^−1^ in about 300 fs after the evolution to the ICT state while the thiophene bending mode at 1221 cm^−1^ get enhanced in the excited state after planarization (see Supplementary Fig. 30). The vibrational cooling dynamics of 2 ps reflects that the evolution of the exciton to ICT and its subsequent cooling is almost retained in the films as well. Therefore, we find that both in solutions and in films it is imperative to tune the thiophene bridge torsion timescales to allow for hot ICT formation.

Our normal mode calculations on the monomer show that the vibrational frequency for thiophene ring torsion is at 125 cm^−1^ (as shown in Supplementary Fig. 9) which of course is expected to be lower in the polymeric backbone. The time-period therefore for the ring rotation should be slower than the expected 270 fs, very much in agreement with our observed timescales of intensity and frequency changes. If such a torsional mode is strongly coupled to the high frequency bends or stretches, then it would also affect their dephasing timescales leading to broadening of the lineshapes in the excited state. It is also important to emphasize here that evolution to the ICT state emerges from the observation of the rate dispersion in cooling dynamics observed by following the frequency shifts. To summarize, our FSRS data therefore strongly hint towards a unified picture that the hot ICT states are generated by low frequency torsional motions coupled to high frequency modes of the donor-π-acceptor backbone. These results motivate innovative experiments to impulsively excite the low-frequency bridge torsions while measuring the FSRS response of the high-frequency modes in order to decipher the mode couplings in an analogous fashion that was reported for intra-molecular CT reactions^32, 54^. In addition, femtosecond optical Kerr effect spectroscopy can also be implemented to demonstrate long-range global modes involved in charge generation process^55^.

## Discussion

**Fig. 6:**
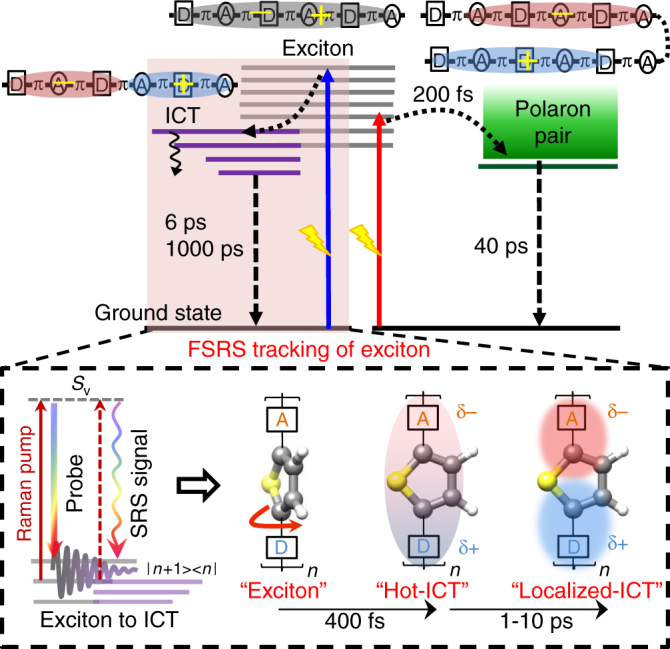
Schematic of the exciton dynamics and its driving molecular coordinates. FSRS measurements directly probe the exciton relaxation via thiophene bridge planarization leading to hot delocalized ICT character, and its subsequent cooling dynamics

Our TA and FSRS data provide unequivocal evidence for formation of the ICT as well as the polaron pair states in the donor-π-acceptor backbone of TDPP-BBT polymer. The presence of both these states can be rationalized by invoking a heterogeneous model which entails two distinct populations of excitons being created with the pump excitation. By pumping at the blue-part of the absorption spectrum (600–700 nm), excitons are generated that are mostly localized on the backbone of one strand while red-excitation from >750–800 nm leads to inter-strand excitons analogous to that proposed by Libai Huang and co-workers for PBDTTT polymers^45, 56^. Figure 6 schematically depicts the fate of these two distinct types of excitons. The inter-strand excitation immediately generates the polaronic states within 100–200 fs both in solutions and films. The polarons are generated with higher yield when the red-absorbing (above 750–800 nm) polymer chains which are packed well are photoexcited. It further gives insight into the possible delocalized character of the red-absorbing population and its photochemistry. While they do indeed form in significant fraction, they are usually too short-lived to be harnessed. The polaronic state decays within 40 ps timescale suggesting that for the charge extraction at the electrode the charge mobility has to be sufficiently high. In addition this short time window for charge extraction provides insight into polymer lengthscale (lower than 2 nm) near the electrode interface which can directly contribute to charges without added acceptors.

The TA measurements with 650 nm excitation suggests that the intra-strand exciton decays with two time component of about 6 ps and 1 ns. The origin of the shorter component is not completely well understood but can possibly arise from fractional recombination of the ICT state before thermalization. The longer component represents the lifetime of the radiative process as supported by the TCSPC measurements. Our FSRS measurements with Raman pump excitation at 816 nm which is post-resonant with the ESA feature at around 1050 nm selectively interrogated the dynamics of the intra-strand exciton. Transient Raman data elaborates our molecular understanding of the exciton relaxation to the intra-molecular charge transfer manifold. Such dynamics is impossible to spectrally resolve using the broad ESA feature of the exciton especially because there is an overlapping polaronic signature at 1350 nm. The timescale of 200–400 fs as deduced from both frequency shifts and intensity changes in the thiophene-centric modes report directly about the RC that drives the relaxation of the exciton. Interestingly this planarization drives the delocalization-induced ICT character of the exciton which is evident from the frequency dynamics of DPP and BBT units within 1 ps timescale. Therefore we believe that the thiophene torsion actually drives the generation of delocalized hot ICT exciton in the backbone of the TDPP-BBT polymer. The rate dispersion in the vibration cooling timescale indicates the thermalization of an ICT state that is partially localized on DPP and BBT chromophores. Our work supports the observation reported by Zhu and co-workers that hot CT excitons even at donor−acceptor interfaces can be generated in organic materials within 200–300 fs and thereby setting the fundamental time limit for charge generation^18^. In future, resonance selective FSRS at the near-IR range will provide the spectral signatures of hot polaron-pair states that are generated in few hundreds of femtoseconds at interfaces.

The thiophene planarization in the excited state of polythiophene homopolymers has been shown previously using both TA^57^ and time-resolved Raman spectroscopy^36^. The planarization is a generic consequence of the structural relaxation after photo-excitation in π-conjugated organic homo-polymers. However in the case of D-π-A backbone the relaxation process can be more complex due to increased conformational degrees of freedom. The direct observation of a thiophene bridge rotation in the D-π-A backbone as probed through our time-resolved Raman snapshots allows for the electronic coupling between the donor and acceptor moiety in the backbone. Remarkably the realization that bridge torsions govern the establishment of the ICT character is an elegant and powerful phenomenology that can be generalized to other low-bandgap polymers for addressing the exciton binding energy problem.

In fact our data also support the hypothesis laid out previously by Scholes and co-workers for the donor−acceptor polymer PCDTBT^26^. The observed red-shift in the diagonal peak and inter-conversion between off-diagonal peaks within 200 fs indicated the possible role of bridge thiophene in CT relaxation although their conclusions were based on computations of the excite state dipole moment. Later, Banerji et al.^30^ demonstrated that PCDTBT monomer has a solvent polarity-dependent emission as well as TA. They showed that solvent rearrangement seem to dominate the relaxation of the ICT state. This process is followed by CT exciton localization in few picosecond timescale. However, the time limit for charge generation is already set at few hundreds of femtoseconds^18, 58, 59^. In the perspective of all the previous work reported on CT exciton generation in organic semiconductors^18, 26^, we find a unified view which suggests that coherent low-frequency backbone torsions which range within the time window of 200–500 fs should dictate the fastest timescale for charge generation. We believe that resonance FSRS measurements on variety of donor-π-acceptor copolymers will enumerate directly the motions that drive the exciton relaxation, polaron pair generation, charge localization and energy hopping through chains. As new technical developments for carrying out FSRS in the NIR region has emerged^60^, the structural dynamics of these key charge intermediates can now be tracked in resonance.

Our results about thiophene bridge torsion implies gating role of bridge vibrations for charge generation in molecular materials. Such a role for vibrations is not surprising since Bakulin et al. were able to demonstrate that excitation of selective vibrational modes can improve the charge transport and photo-response in organic optoelectronic systems^61^. In addition recently, Delor et al. have shown the significance of selective vibrational modes in charge transfer in donor–bridge–acceptor assemblies^62, 63^. The ET step here was tracked with time-resolved IR measurements and showed important role of the CO bridge in a hetero-metallic transition metal complex. The ground state planarity of donor-acceptor backbone along with the polymer persistence length plays a critical role in determining both the absorption cross-section^64^ and the photoinduced exciton dynamics^12^, thereby affecting the charge generation yields^65^. Most of the contemporary donor-π-acceptor polymers which show high power conversion efficiency have conjugated π-bridges that dynamically influence the coupling between donor and acceptor units^12, 41, 66^. Our results here clearly demonstrate that in TDPP class of donor-π-acceptor polymers the bridge torsion timescale plays an important role and sets a benchmark for charge generation. Significantly the ICT state generation was reported by Provencher et al. in the PCDTBT set of polymers to be slightly slower which possibly allows for hot delocalized charge separation in polymer:fullerene blends^37^. In fact, the large delocalization length of the charge separated state as reported by them supports that with a slower ICT leads to generation of hot CS states that can be accessed at the interfaces. Synthetically tuning the thiophene bridge torsion timescale by introducing non-covalent interaction between the bridge and other structural parts of the polymer will help  optimize hot CT generation and relaxation rates leading to solar cell designs with higher *V*_oc_ and tunable *J*_sc_. Therefore, rational designs of donor-π-acceptor polymers based on our proposed dynamical framework will not only allow for long-range separation between donor and acceptor moieties but also gate the timescale of the charge transfer process by appropriate choice of π-bridge.

In summary, we have probed the primary reactive motion that drives the exciton relaxation-induced ICT in the donor-π-acceptor backbone of TDPP-BBT polymer. Broadband TA spectroscopy on the TDPP-BBT polymer in solution reveals the simultaneous presence of both a long-lived (about 1000 ps) exciton and short-lived polaron pairs (about 40 ps) due to conformational heterogeneity. An ultrafast evolution of strongly bound Frenkel excitons to weakly bound polaron pairs does occur in 100–200 fs for polymer chains that have strong inter-strand coupling. We dissected the exciton and polaron pair heterogeneity by selectively probing the exciton population using resonance-enhanced FSRS. The transient structural evolution for exciton relaxation dynamics in TDPP-BBT was characterized by enhanced Raman activity of the 1228 cm^−1^ C−H bending mode along with red-shifting of the C=C stretching of bridge thiophene. The rise in the Raman cross-section within 400 fs indicates sub-picosecond torsional relaxation of the exciton through thiophene bridge planarization. The observed cooling in the donor and acceptor vibrational modes by 1–10 ps underlies the fundamental nature of the ICT exciton that is created after excitation. Thus the generation of the hot delocalized ICT state by a thiophene bridge motion in about 400 fs provides phenomenological insight into the charge generation and relaxation dynamics in generic donor-π-acceptor polymers. In order to engineer novel polymeric materials for efficient photovoltaics, design principle should imbibe optimization of backbone torsion timescales especially the π-bridge motions which gate the formation of the ICT state, the precursor to free charges.

## Methods

### Materials

For majority of experiments described in the manuscript the chemicals were obtained from commercial sources, and used without further purification. The starting materials for synthesis of low bandgap TDPP-BBT polymer, i.e., 2,5-bis(2-octyldodecyl)-3,6-bis(5-(4,4,5,5-tetramethyl-1,3,2-dioxaborolan-2-yl)thiophen-2-yl)pyrrolo[3,4-c]pyrrole-1,4 (2H, 5H)-dione (M1) and 4,8-bis(dodecyloxy)benzo[1,2-b:4,5-b′]dithiophene (M2) were synthesized following the procedures described in the literature^67, 68^. Solvents were dried and distilled out before being used for the synthesis. The ^1^H -nuclear magnetic resonance (NMR) spectra of the synthesized polymer were recorded on a Bruker Advance NMR spectrometer at 400 MHz frequency. CDCl_3_ along with TMS were purchased commercially and used for NMR measurements. Average molecular weight (*M*_n_ = 9.035 kDa, *M*_w_ = 28.42 kDa) were determined by Gel permeation chromatography against monodisperse polystyrene standards. Tetrahydrofuran was used as eluent with a flow rate of 0.5 ml per min. Experimental procedure and synthetic methods are described in Supplementary Note 1.

### Steady-state spectral measurements

The steady-state absorption measurements were carried out in a commercial JASCO V-670 spectrophotometer. A quartz cuvette with 2 mm path length was used to keep the absorbance below 1 OD unit. All steady-state emission spectra were measured in a Horiba Jobin-Yvon SPEX Fluorolog-3 spectrofluorometer using a 2 mm path length quartz cuvette. Fluorescence spectra were corrected for the spectral sensitivity of photomultiplier. The slit widths were adjusted to keep the detection in the linear range.

### Film preparation

Three milligrams of the neat polymer was dissolved in 200 μl of chlorobenzene and heated at 60 °C for 3 h. After cooling the solution to room temperature, 20 µl of the solution was spin coated for 1 min at 2000 rpm with an acceleration of 200 rpm per second on top a 1 mm thick quartz substrate that was cleaned sequentially by detergent, de-ionized water, acetone and finally using isopropanol.

### Time-resolved fluorescence measurements

Time-resolved emission experiments were performed using a picosecond TCSPC technique. Frequency doubled (532 nm, 400–600 mW) output of CW, mode-locked Nd-YAG laser (Spectra Physics series 3000) was used to synchronously pump cavity dumped, mode-locked, picosecond Rhodamine 6G dye laser (570−630 nm) (Spectra Physics) to generate excitation pulse with pulse width of typically 4–10 ps and repetition rate of 800 kHz. The fluorescence photons were collected at the count rate of below 8000 counts per second. The dye was continuously circulated to avoid any photobleaching. TDPP-BBT samples were excited at 630 nm. Fluorescence decay traces at 690 nm were collected using a microchannel plate photomultiplier (model R2809; Hamamatsu Corporation) that is coupled to a single photon-counting setup. The IRF at 630 nm was obtained by using a colloidal solution of dried coffee whitener. The average FWHM of the IRF from multiple measurements was about 90 ps. A 650 nm long pass filter was used for the fluorescence measurements. All the emission measurements were carried out at magic angle (54.7°) to eliminate any contribution from fluorescence anisotropy decay.

### Femtosecond transient absorption measurements

The detailed description of the pump-probe set up has been mentioned elsewhere^69^. Briefly, fundamental output from an 80 MHz repetition rate femtosecond oscillator (Coherent Micra-5 modelocked Ti:Sapphire Laser system) with bandwidth of about 100 nm and power of 5 nJ per pulse is amplified using Coherent Legend Elite^®^ regenerative amplifier. The output of the amplifier is about 4 mJ per pulse, with a repetition rate of 1 kHz and near 30 fs pulse width. Part of this amplified pulse was used to generate the 650 and 700 nm pump pulse using an optical parametric amplifier Coherent OPeraASolo^®^. The pump pulse energy was reduced to 0.25 mW (focal spot of 270 μm inside cuvette) in order to minimize the photo-damage. The probe pulse was chosen from portion of the white-light continuum that was generated using a 2 mm thick sapphire crystal. The NIR regime (800–1300 nm) of the probe continuum was spectrally cleaned using a 850 nm long pass filter. After passing through the sample, probe is finally dispersed by Helios^®^ spectrograph onto a 297 pixel imaging element. The pump and probe pulses were focused and spatially overlapped on the flowing sample contained inside a cuvette with 0.5 mm quartz glass window. The IRF with 650 nm excitation pump and NIR probe was determined to be about 90 fs using an optical Kerr arrangement. All pump-probe measurements were done in the flowing condition with the polymer solution driven by a peristaltic pump. In order to refurnish fresh sample before every shot the flow rate was suitably adjusted. For the film measurements, the pump and probe pulses were loosely focused while spatially overlapped directly on the film sample kept inside a anaerobic chamber under N_2_ atmosphere. Pump pulse energy used was 0.15 mW for the film measurements. The film pump-probe measurements were repeated at the same spot as well as at different film spots to check reproducibility.

### Femtosecond stimulated Raman scattering

Femtosecond stimulated Raman scattering (FSRS) can be used to get fluorescence background-free coherent Raman signal. Here a combination of narrow bandwidth Raman pump centered at 816 nm (measured using Ocean optics spectrometer) along with a broadband femtosecond NIR (850–1300 nm) probe pulse stimulates the coherent Raman scattering process from the sample. The femtosecond NIR probe pulse was generated after focusing the fundamental onto a sapphire crystal, and was compressed subsequently using a home-built two-prism compressor set up. The narrow bandwidth Raman pump pulse was generated from the broadband femtosecond fundamental pulse using a grating filter configuration that was previously reported^70^. Both Raman pump and probe pulses are overlapped in time and space on the sample to get SRS signal. The cyclohexane SRS signal is shown in Supplementary Fig. 37. The spectral resolution was limited by the Raman pump pulse which was set at about 3 ps (Supplementary Fig. 38). The solution measurements were carried out in a flow cuvette as described previously in the TA measurements section. FSRS measurements were carried out on TDPP-BBT film in a metal chamber (with 1 mm quartz glass window) filled up with N_2_ gas. The Raman pump power was reduced to 1 mW for optimal signals without photodamage. The transmitted probe beam from the sample was dispersed using Acton SP 2300 spectrograph and spectra were recorded with a computer-controlled Pixis^®^ 100F CCD detector (Princeton Instruments, Roper Scientific). Shot-to-shot detection of the pump-on vs pump-off spectra using lock-in detection provided fast accumulation of data with minimum spectral variations. Each ground state and excited state spectrum was accumulated for 10 s and identical experimental conditions were maintained for all samples. The detector was calibrated using the CCD-detected stimulated Raman spectrum (about 10 cm^-1^ spectral resolution) of cyclohexane collected under identical conditions. The 650 nm actinic pulse was used to get the excited state SRS signal. Time resolution is dictated by the actinic pump and probe pulse timing which comes around 70 fs (Supplementary Fig. 36). We record SRS signal with and without actinic pump and subtract them. The details of the FSRS signal processing is described in the Supplementary Notes 4–5. The sample integrity of both film and solution is checked by recording ground state Raman signal before and after the excited state measurements as shown in Supplementary Figs. 33 and 39.

### Computational methodology

Optimization of the molecular structures (see Supplementary Fig. 7) and vibrational Raman spectra (see Supplementary Figs. 8–9) calculations for the optimized structures were performed by means of DFT with the Becke’s 3 parameters and the Lee–Yang–Parr’s nonlocal correlation functional (B3LYP) with a 6–31+ G(d) basis set, obtained using GAUSSIAN09^®^ package. For comparing the computationally predicted Raman with that from experiments, all the calculated Raman frequencies were scaled by a factor 0.97 due to the correction of the harmonic approximation. The intensities of the Raman bands were estimated by the Gaussian package calculating the differential Raman scattering cross-section.

### Data availability

The data sets generated and analyzed during the current study are available from the corresponding authors upon request.

## Electronic supplementary material

Supplementary Information
