# Author Correction: Ultrafast bridge planarization in donor-π-acceptor copolymers drives intramolecular charge transfer

**DOI:** 10.1038/s41467-017-02574-1

**Published:** 2018-01-03

**Authors:** Palas Roy, Ajay Jha, Vineeth B. Yasarapudi, Thulasi Ram, Boregowda Puttaraju, Satish Patil, Jyotishman Dasgupta

**Affiliations:** 1 Department of Chemical Sciences, Tata Institute of Fundamental Research, 400005 Mumbai, India; 2 Department of Nuclear and Atomic Physics, Tata Institute of Fundamental Research, 400005 Mumbai, India; 3 Solid State and Structural Chemistry Unit Indian Institute of Science, 560012 Bangalore, India

Correction to: *Nature Communications* 10.1038/s41467-017-01928-z, published online 23 November 2017

The original version of this Article omitted the following from the Acknowledgements:

“Satish Patil thanks Department of Science and Technology, New Delhi, India for a Swarnajayanti fellowship.”

This has now been corrected in both the PDF and HTML versions of the Article.

