# Supplementary material for: Ultrafast bridge planarization in donor-π-acceptor copolymers drives intramolecular charge transfer
Source: Nat Commun. 2017 Nov 23;8:1716. doi: 10.1038/s41467-017-01928-z (PMC5700982; doi:10.1038/s41467-017-01928-z)
Supplement: Supplementary file 1 — Supplementary Information [file 41467_2017_1928_MOESM1_ESM.pdf]

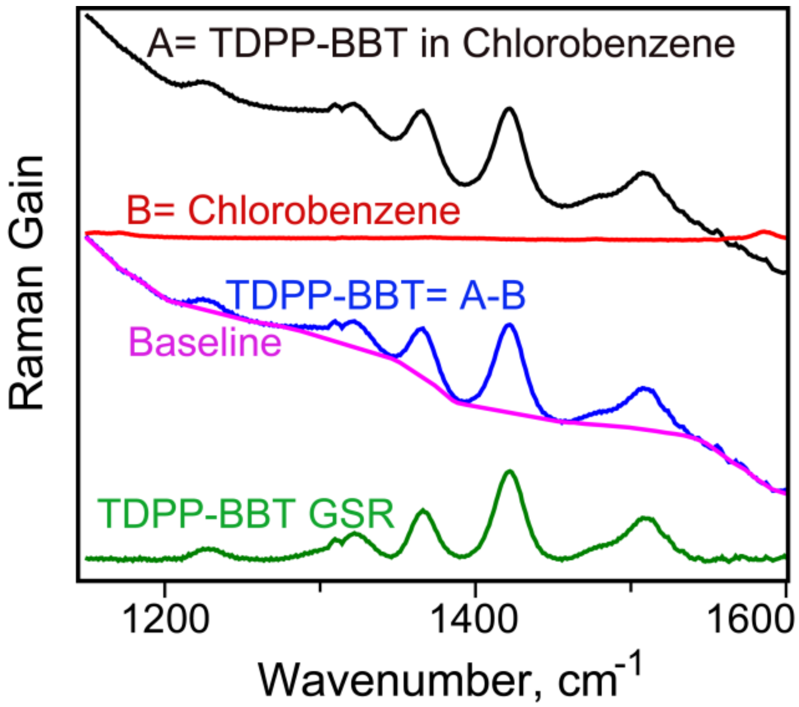

Supplementary Figure 1. Ground state stimulated Raman (GSR) signal processing for TDPP-BBT copolymer.

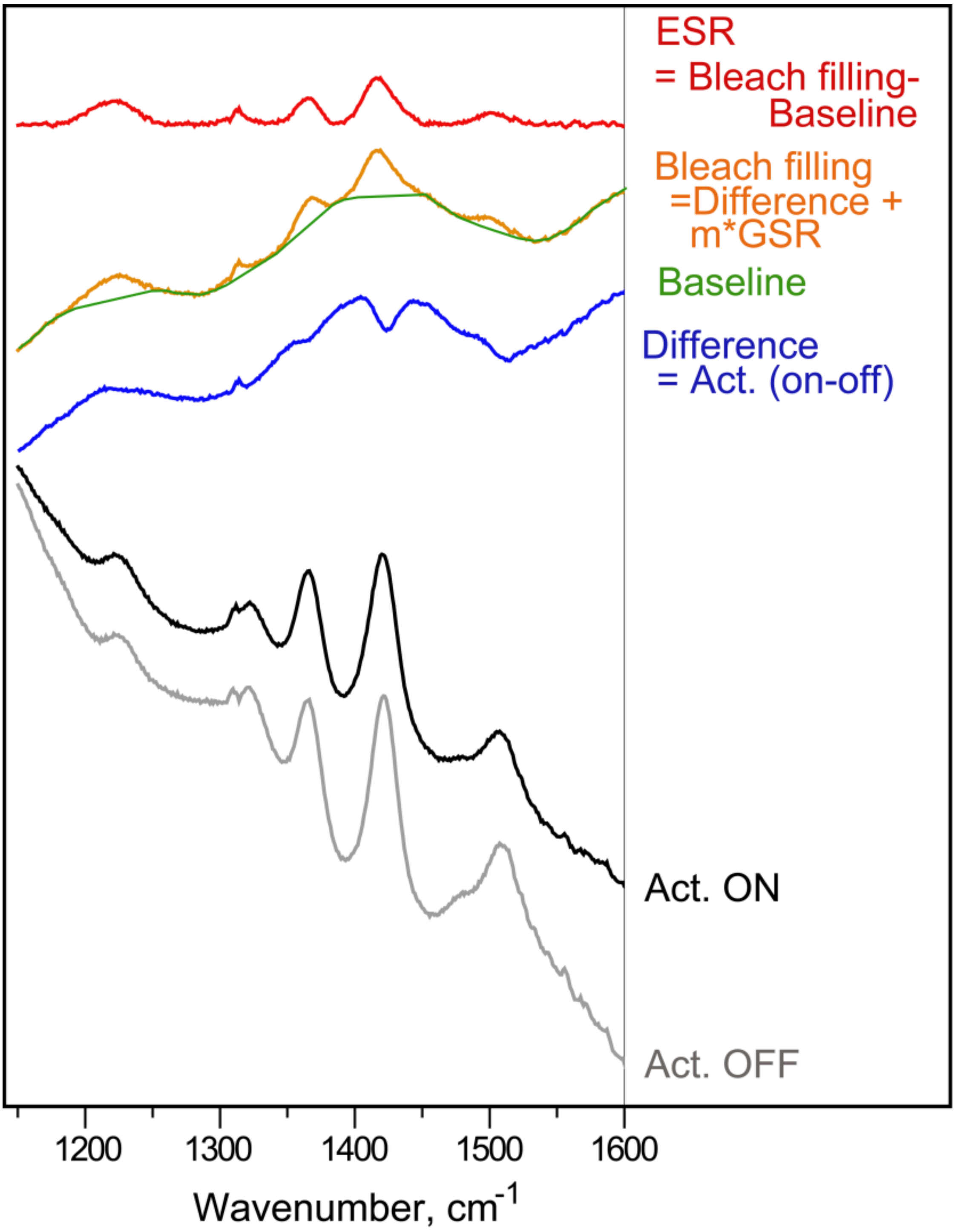

Supplementary Figure 2. Excited state stimulated Raman signal (ESR) processing for TDPP-BBT copolymer.

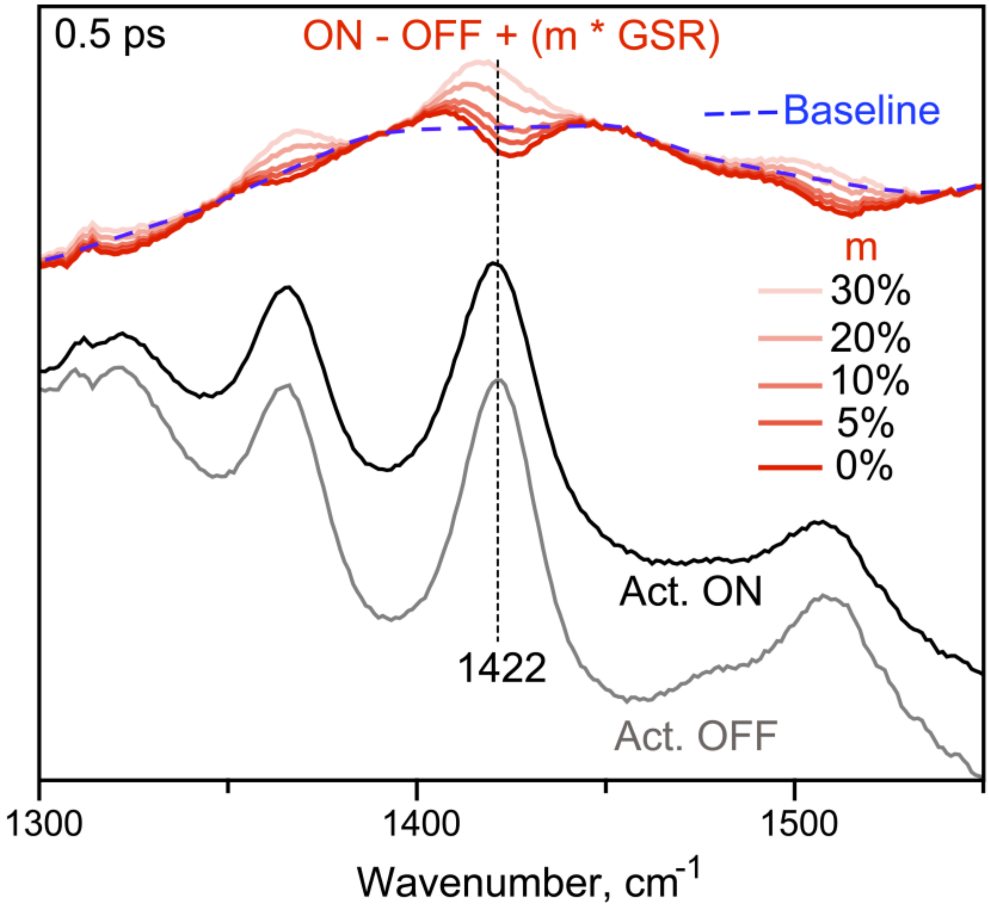

Supplementary Figure 3. Metric for bleach filling of the difference spectra (i.e. ON minus OFF) at 0.5 ps. Different fraction (m) of the ground state Raman (GSR) has been added with difference spectra to result into the excited state Raman (red spectra). The blue dotted line represents baseline.

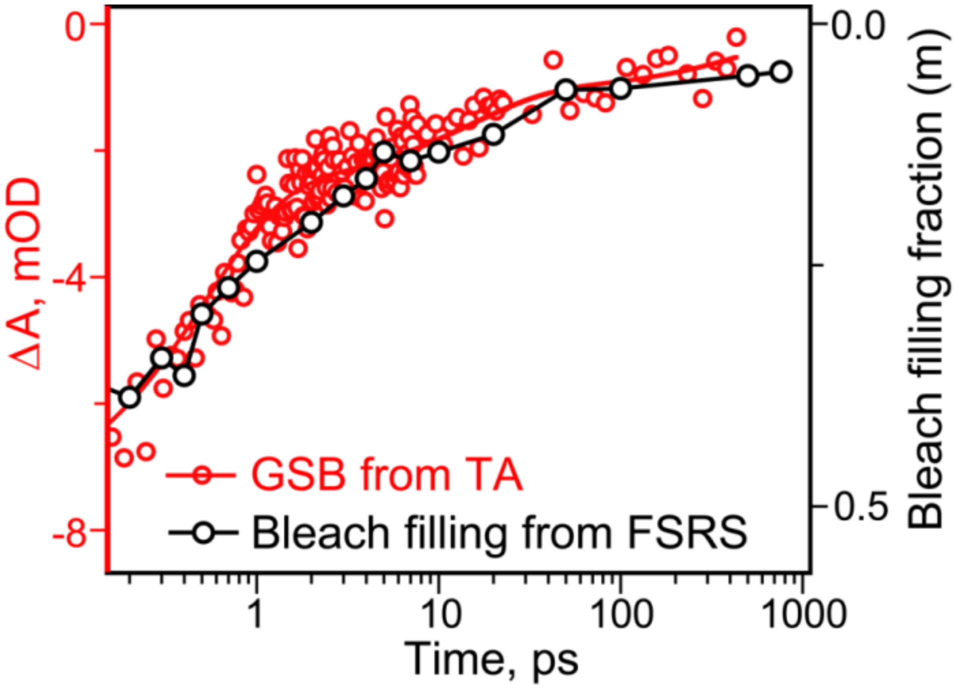

Supplementary Figure 4. Plot of the metric "m" with the ground state bleach obtained from the Transient absorption experiment of the sample with Raman pump-on. The Bleach filling metric (black) closely matches the GSB dynamics (red) assuring that our excited state Raman spectra intensities are justified.

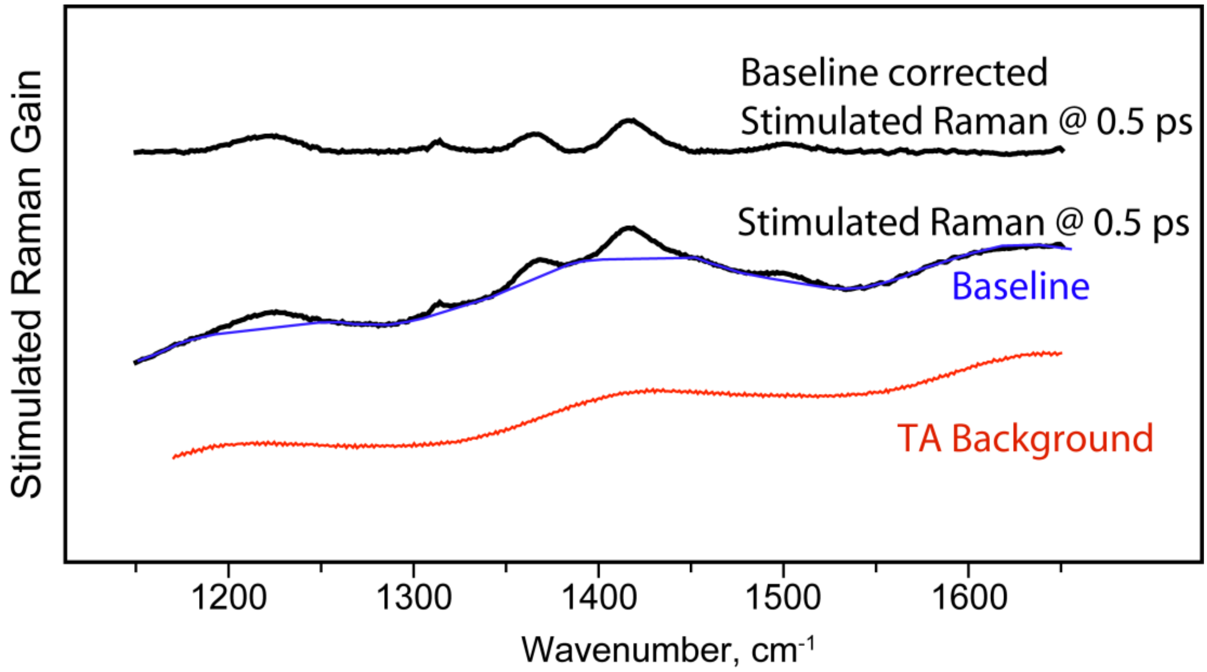

Supplementary Figure 5. The transient absorption (TA) data at 0.5 ps time delay recorded in CCD is plotted with the stimulated Raman signal (black) with baseline (blue). This shows that the background in the FSRS is coming due to TA background.

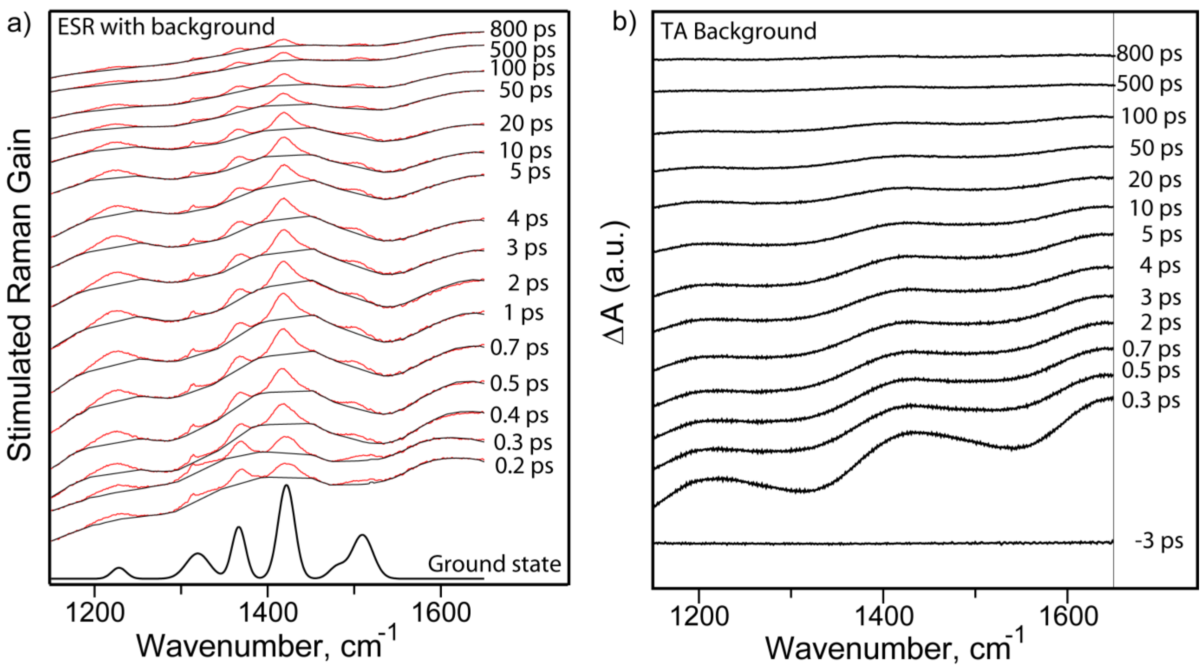

Supplementary Figure 6. a) Excited state stimulated Raman data (red) at different time delays with the baseline drawn (black). b) The transient absorption (TA) datasets at different time delays recorded in CCD. This shows that the background in the FSRS is coming due to TA background.

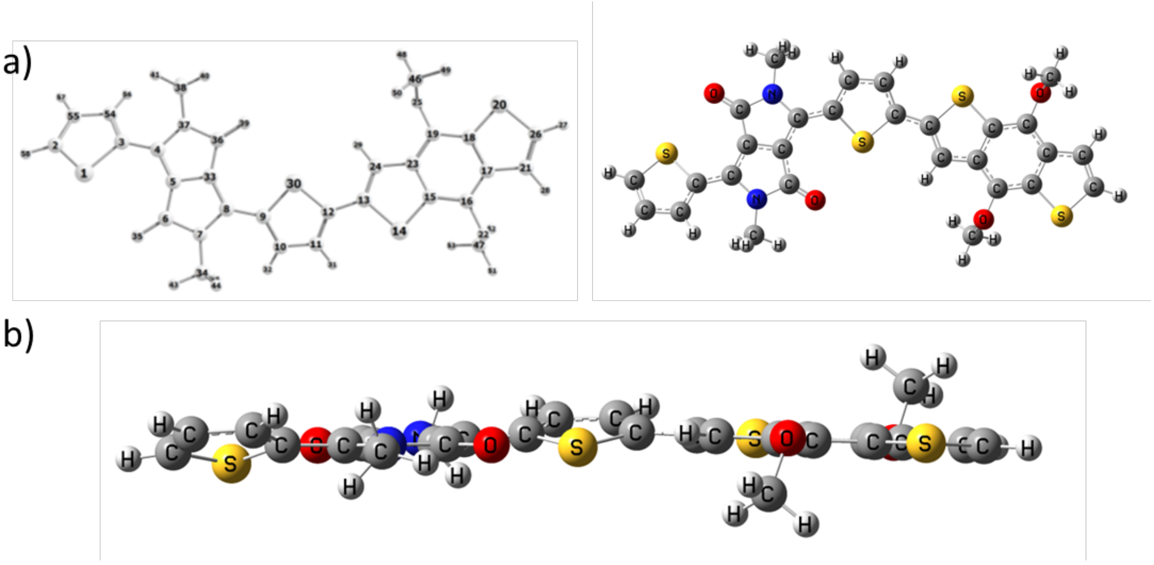

Supplementary Figure 7. a) Side and b) top view of the optimized structure of TDPP-BBT repeating unit.

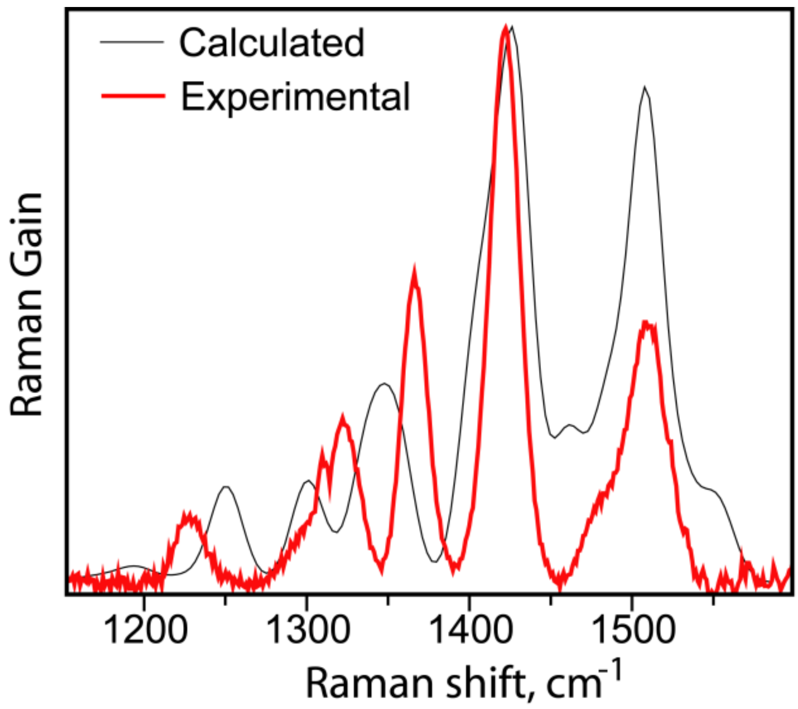

Supplementary Figure 8. Assigning Raman frequencies based on DFT calculation on the optimized TDPP-BBT repeating unit.

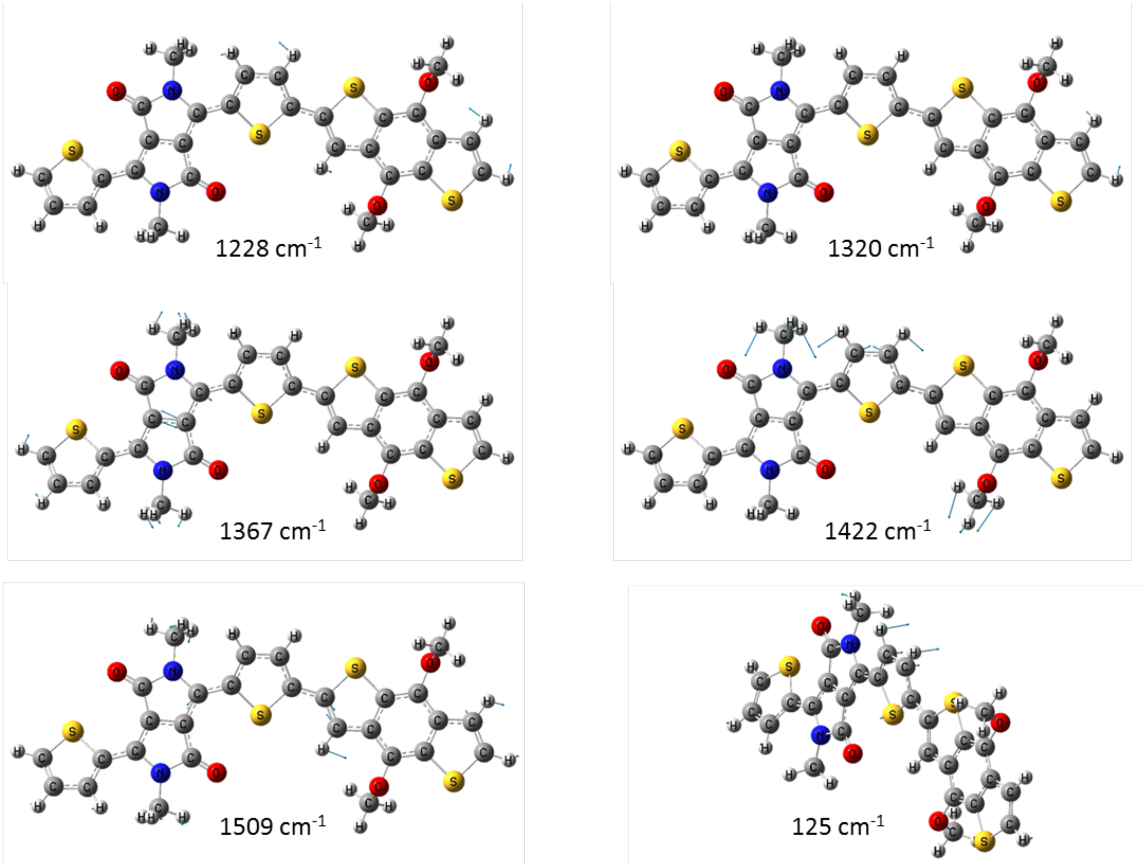

Supplementary Figure 9. DFT calculation (B3LYP) showing optimized structure and Raman frequencies.

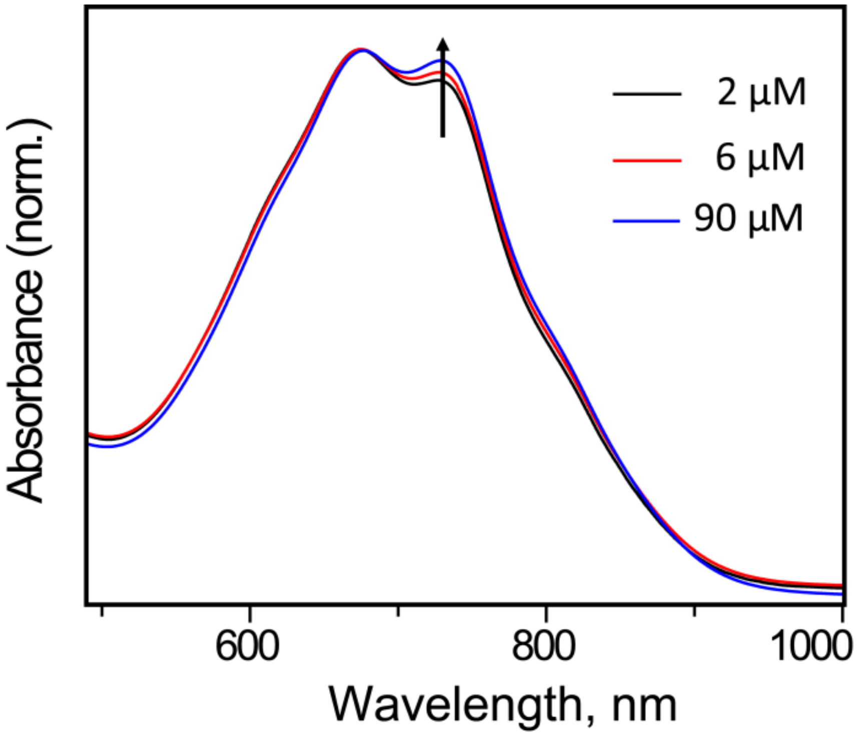

Supplementary Figure 10. Concentration dependent steady-state absorption spectra of TDPP-BBT copolymer in chlorobenzene.

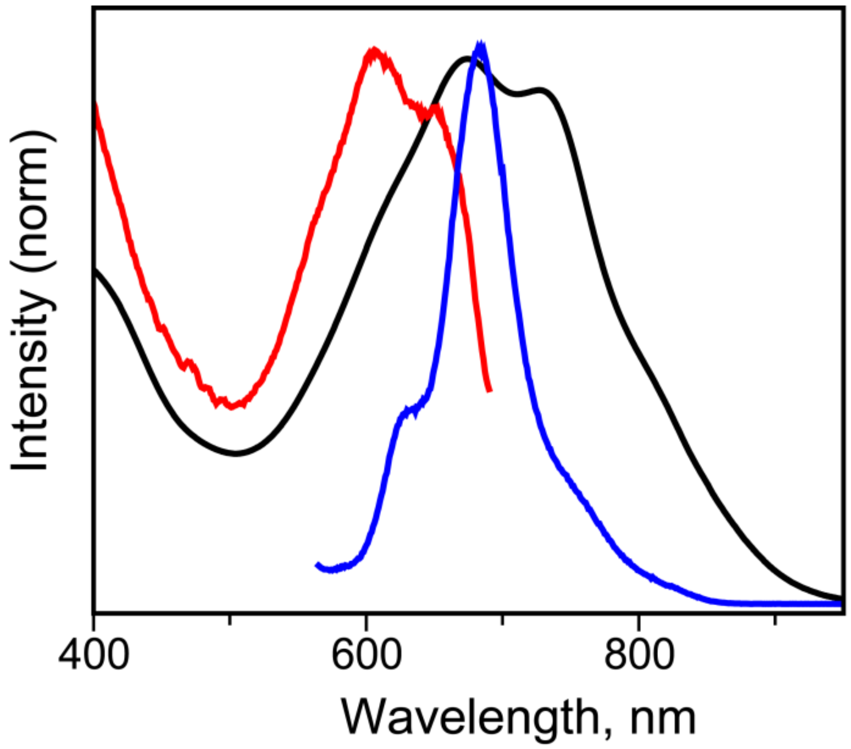

Supplementary Figure 11. Steady-state absorption (black), emission (blue) (at 555 nm excitation) and excitation (red) (at 700 nm emission) spectra of 0.1 μM TDPP-BBT copolymer in chlorobenzene (CLB).

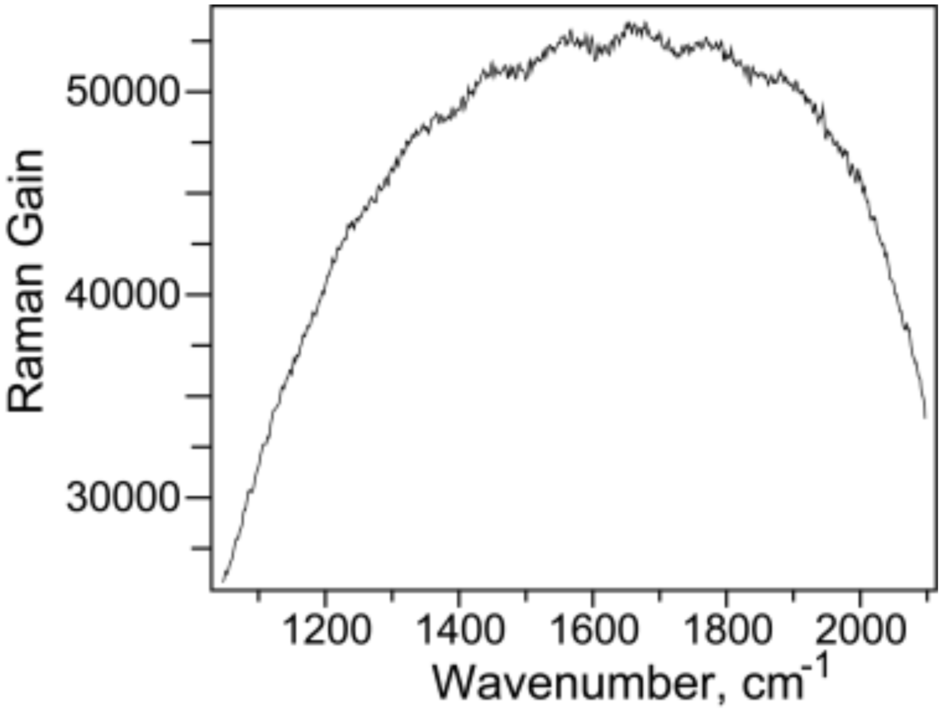

Supplementary Figure 12. Steady-state Raman signal of TDPP-BBT copolymer in CLB (WITEC confocal Raman microscope). 532 nm is the excitation wavelength. It shows a very broad background due to fluorescence.

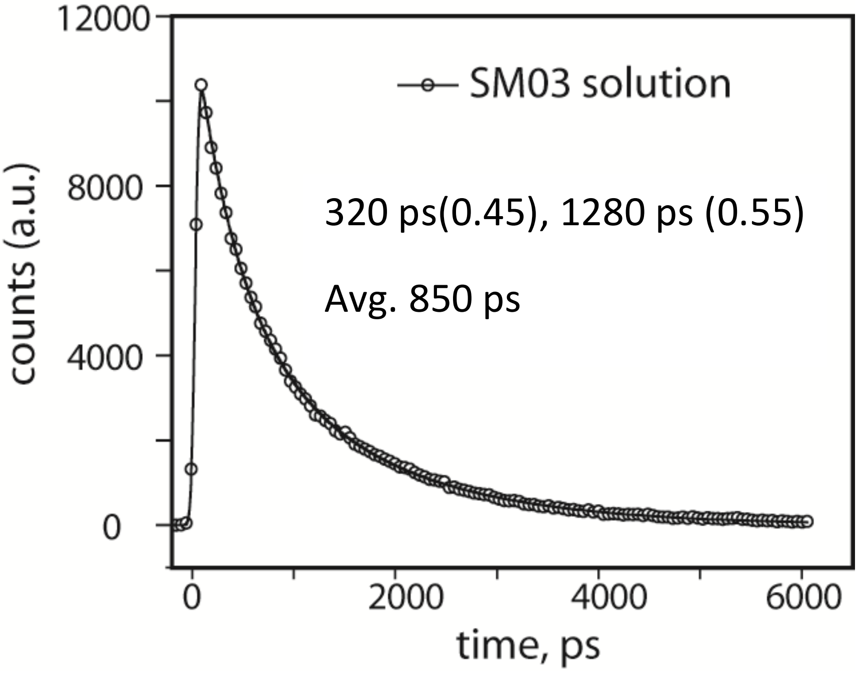

Supplementary Figure 13. TCSPC lifetime measurement of pristine TDPP-BBT copolymer in CLB with λ_{ex} at 630 nm and λ_{em} collected at 690 nm.

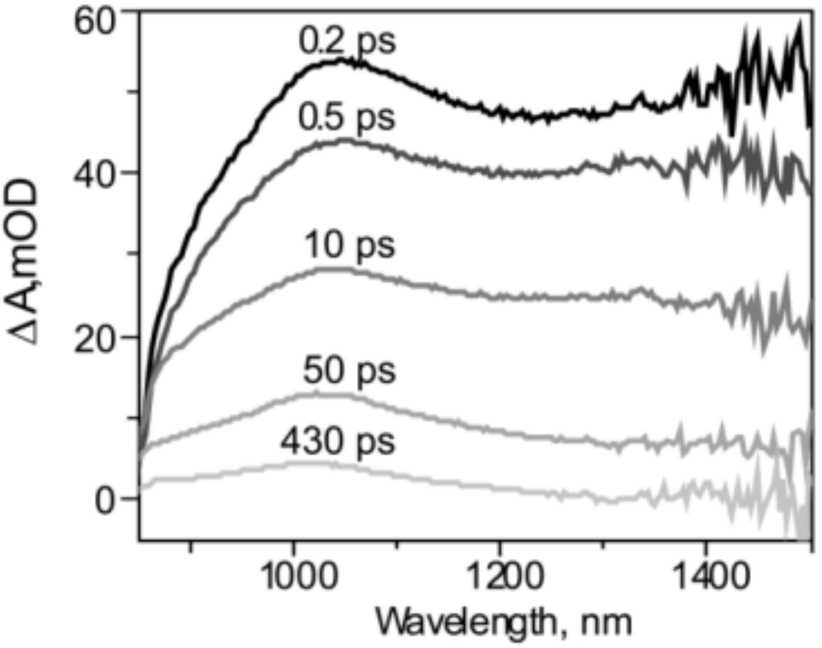

Supplementary Figure 14. Transient absorption of 90 μM TDPP-BBT copolymer in CLB with actinic pump at 650 nm 0.25 mW. The spectral evolution at different pump-probe delays has been plotted.

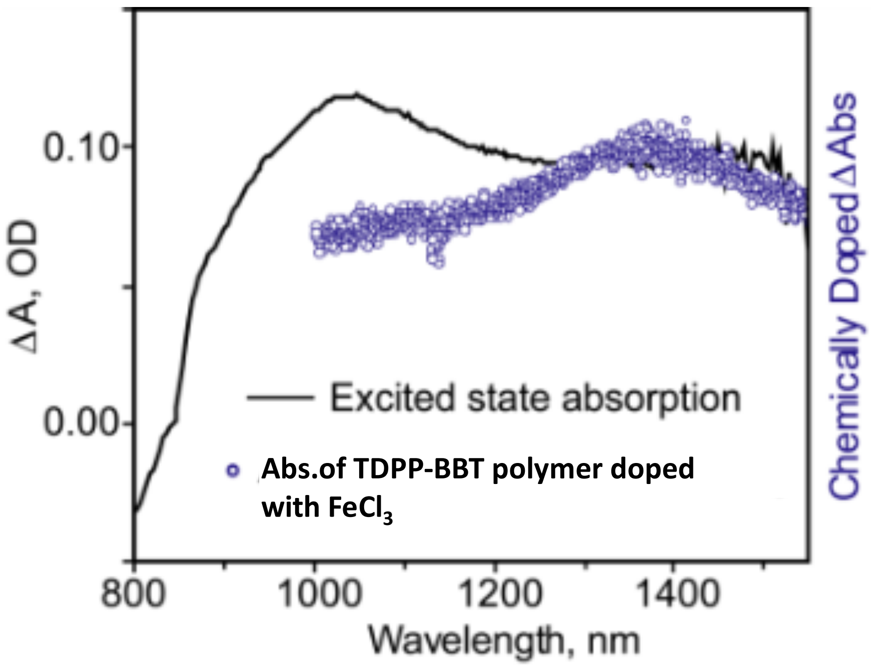

Supplementary Figure 15. Excited state absorption spectrum at 1 ps (black) has been overlapped with chemically oxidised (by $FeCl_3$) TDPP-BBT copolymer absorption spectrum (blue). Both the absorption spectra matches near 1350 nm.

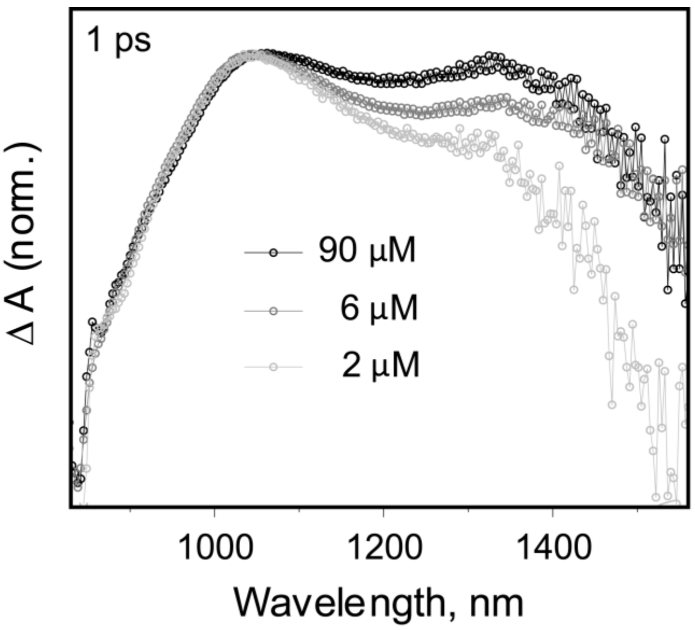

Supplementary Figure 16. Transient absorption spectra of 2, 6 and 90 μM of TDPP-BBT copolymer in chlorobenzene with actinic pump at 650 nm (0.25 mW). Transient spectral traces at 1 ps have been normalized for comparison.

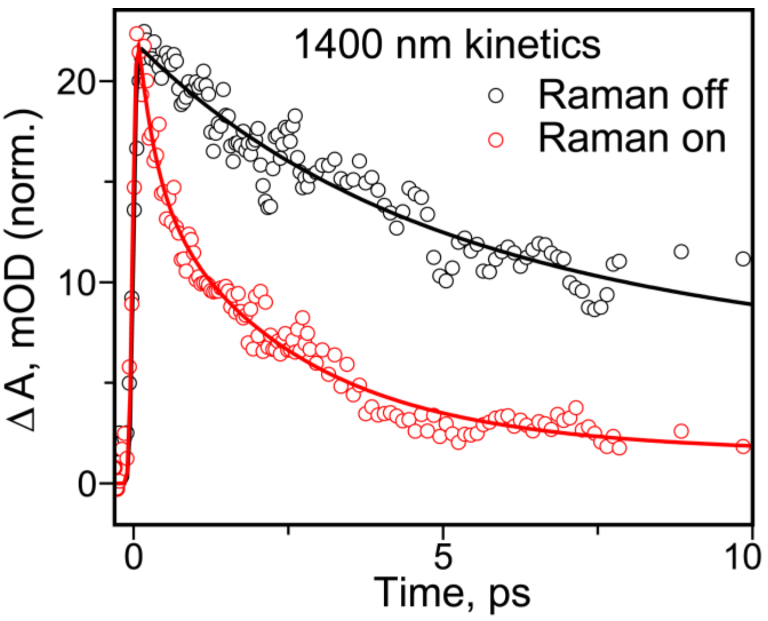

Supplementary Figure 17. TA kinetics at 1400 nm of TDPPBBT in CLB at 650 nm pump in presence and absence of 1 mW 816 nm Raman pump. It shows a fast dump (with around 0.3 ps time constant) of polaron pair population in presence of Raman pump.

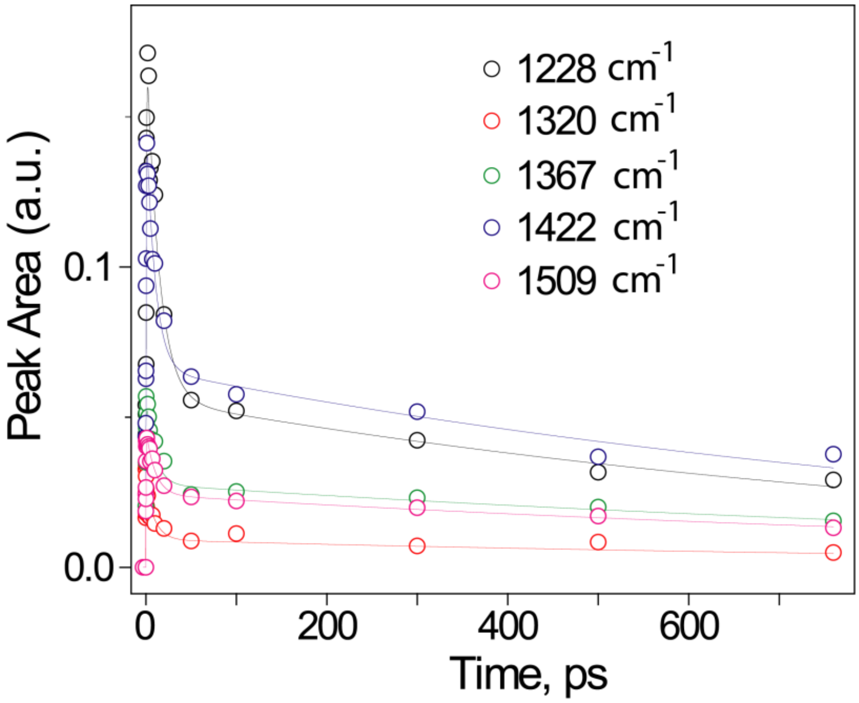

PeakArea	Rise (ps)	Decay1 (ps)	Decay2 (ps)
1228 cm^{-1} peak	0.5±0.2	14	1100
1320 cm^{-1} peak	-------	15	1100
1367 cm^{-1} Peak	0.35±0.13	12	1100
1422 cm^{-1} Peak	0.3±0.1	10	1100
1509 cm^{-1} peak	0.29±0.05	12	1100

Supplementary Figure 18. Peak area kinetics for all the five modes as obtained from FSRS measurement. See Supplementary Figure 20 for rise time plot in the initial time scale.

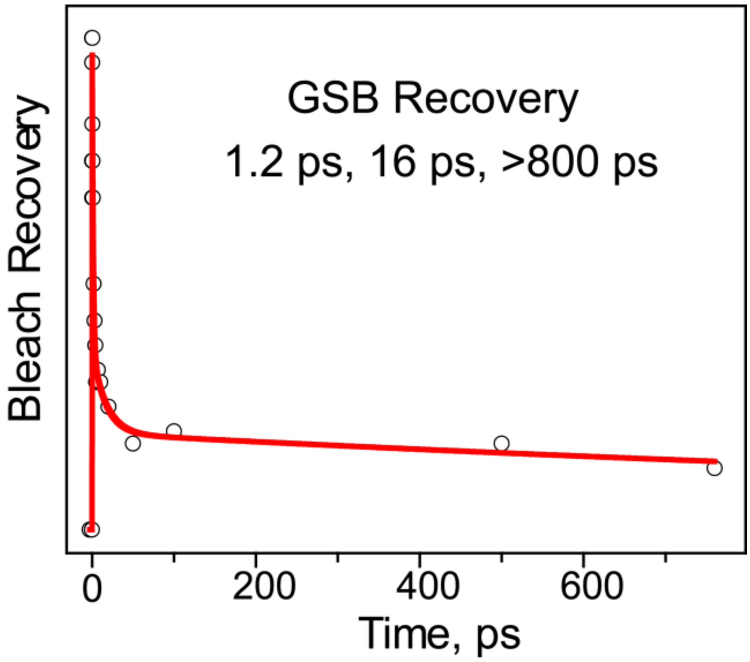

Supplementary Figure 19. Bleach recovery kinetics for FSRS data analysis showing a long lived component.

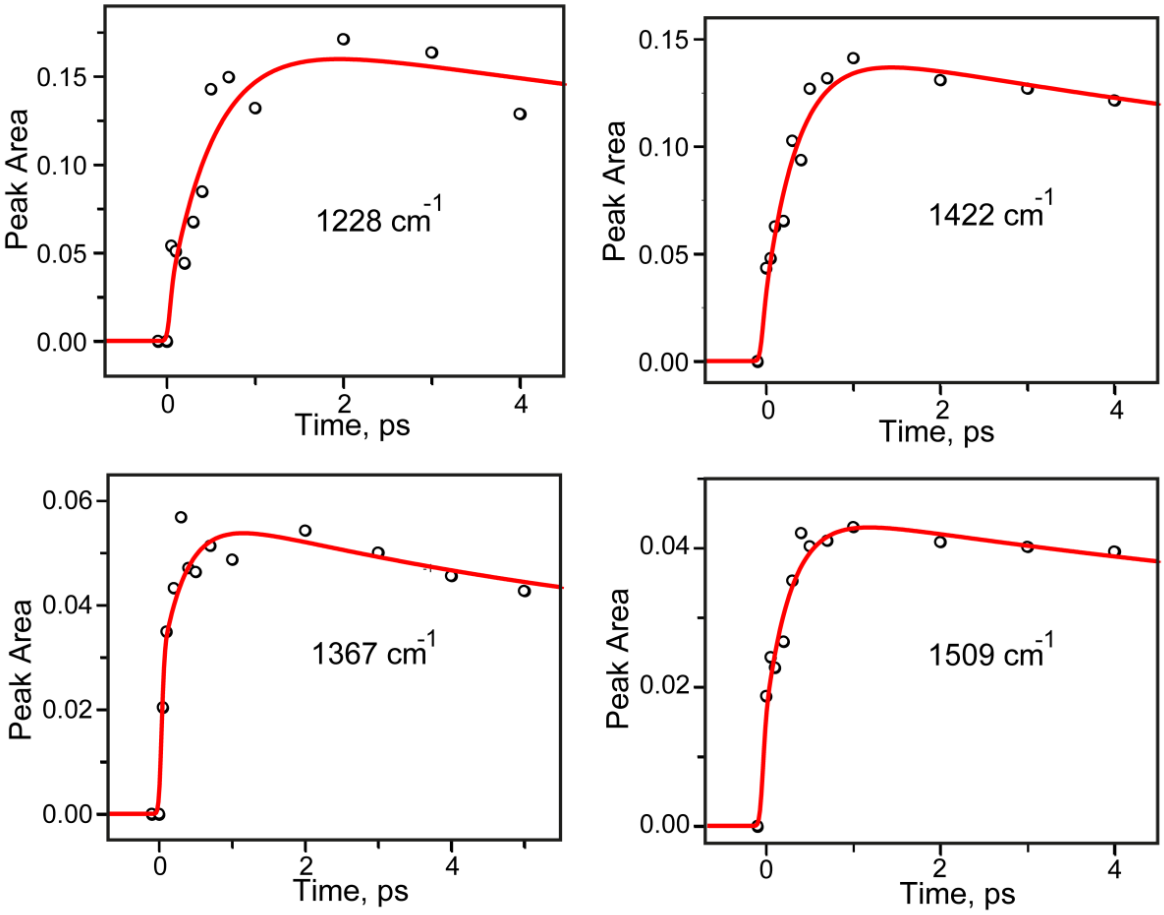

Supplementary Figure 20. Peak area rise kinetics for 1228, 1367, 1422 and 1509 cm^{-1} mode as obtained from FSRS measurement.

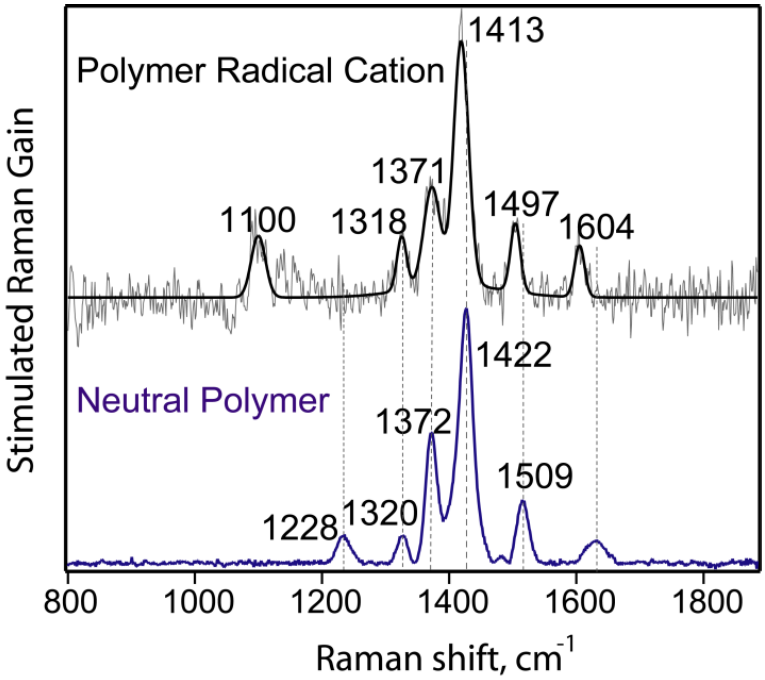

Supplementary Figure 21. Comparison of ground state stimulated Raman spectra of neat TDPP-BBT copolymer solution and its chemically oxidized product ($FeCl_3$ was used as an oxidizing agent).

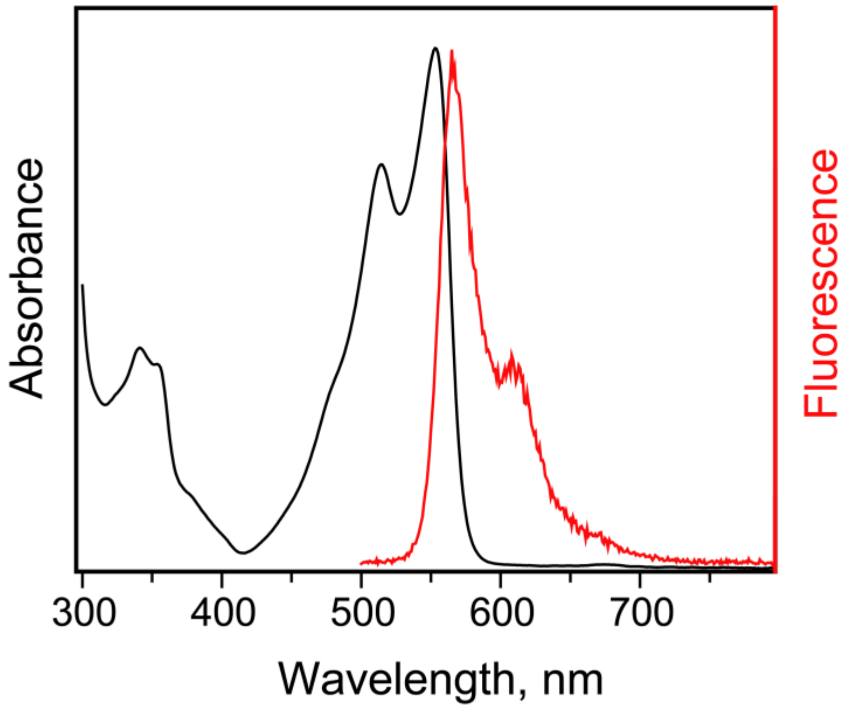

Supplementary Figure 22. Absorption and emission of thiophene-diketopyrrolopyrrole-thiophene (TDPP) monomer in CLB.

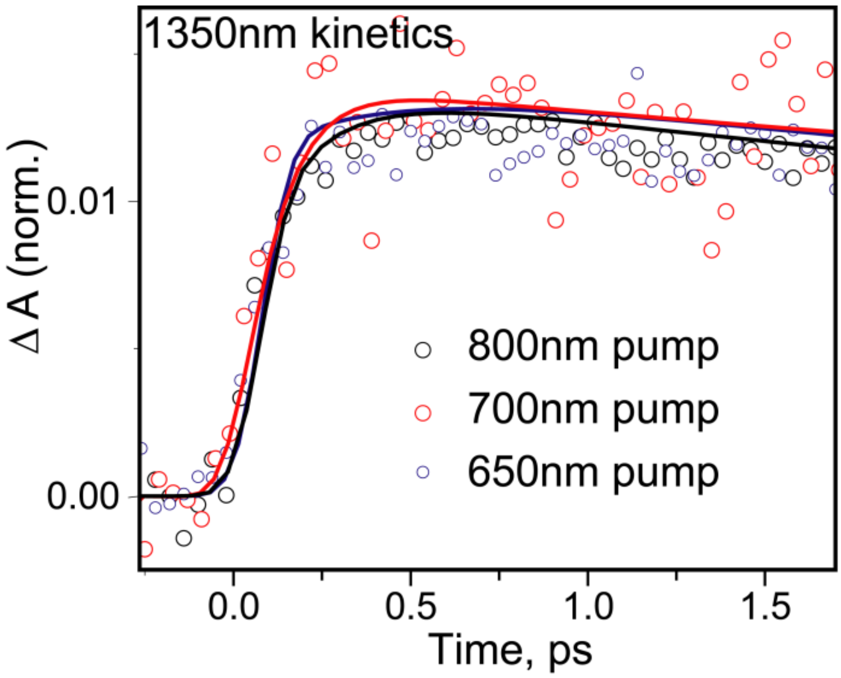

Pump wavelength	Rise time @ 1350 nm
650 nm	0.23±0.07 ps
700 nm	0.15±0.05 ps
800 nm	0.19±0.05 ps

Supplementary Figure 23. Pump wavelength dependent transient absorption. The kinetics at 1350 nm has been plotted for various pump excitations showing rise of 1350 nm kinetics. The ultrafast rise time values are plotted in the table.

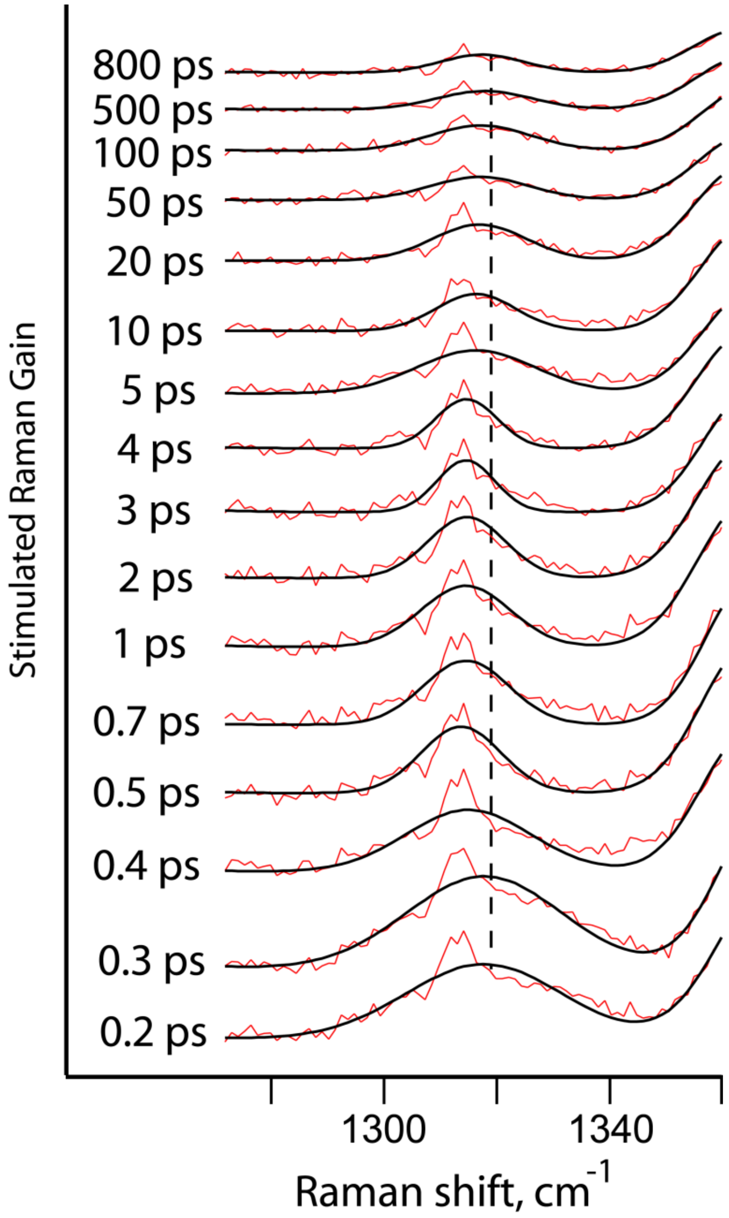

Supplementary Figure 24. Gaussian peak fit for the 1320 cm^{-1} peak has been plotted. This is consistent with a different dataset as shown in Supplementary Figure 25 which does not contain the instrument artifact.

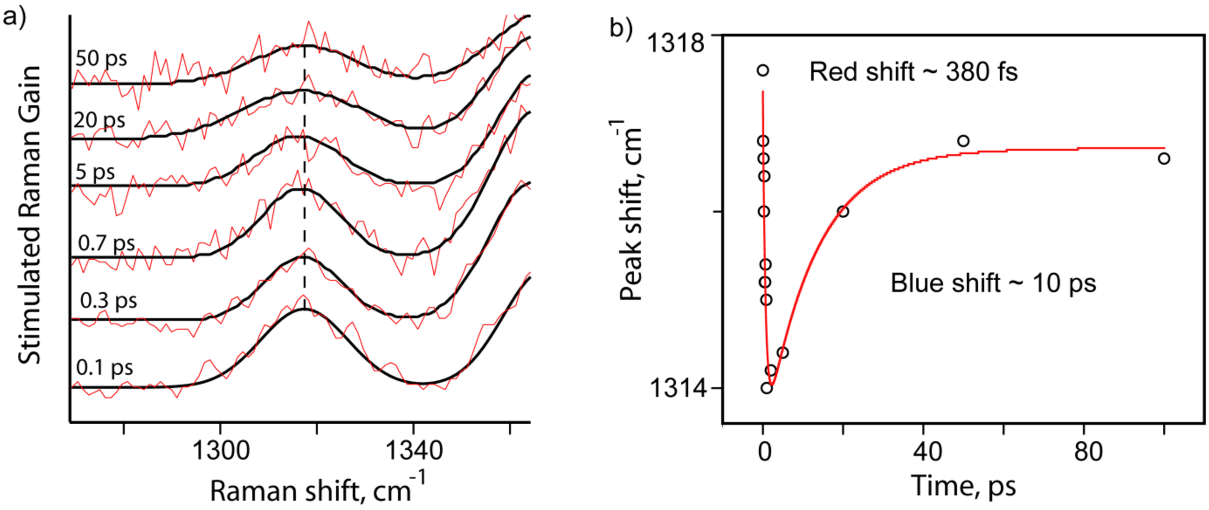

Supplementary Figure 25. a) 1320 cm^{-1} peak shift for FSRS obtained from a different data set that does not have the instrument artifact. Thus it is used to determine the peak shift. b) The 1320 cm^{-1} peak shift shows similar trend to that of main FSRS dataset.

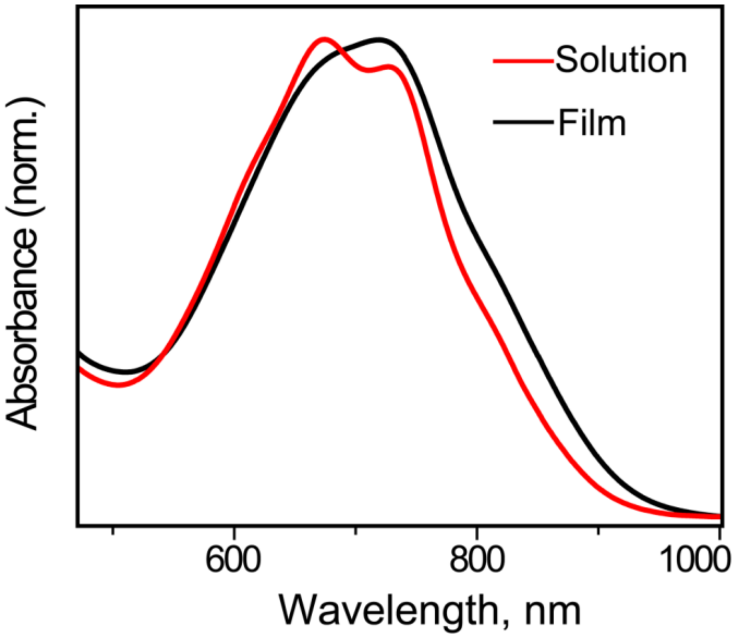

Supplementary Figure 26. Comparison of the steady-state absorption spectra of TDPP-BBT copolymer in chlorobenzene solution and in dried film.

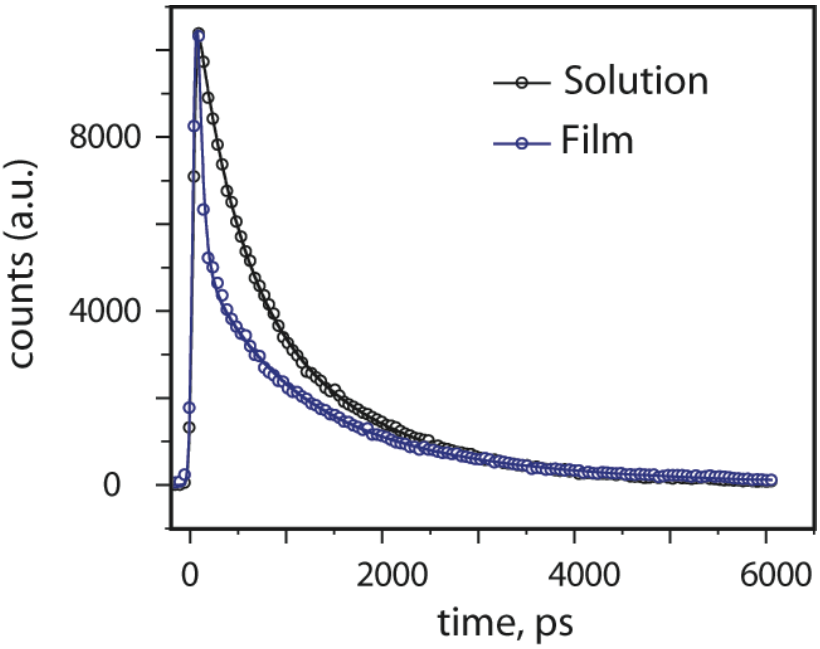

Supplementary Figure 27. Comparison of TCSPC lifetime measurement of pristine TDPP-BBT copolymer in chlorobenzene and dried film with λ_{ex} at 630 nm and λ_{em} collected at 690 nm.

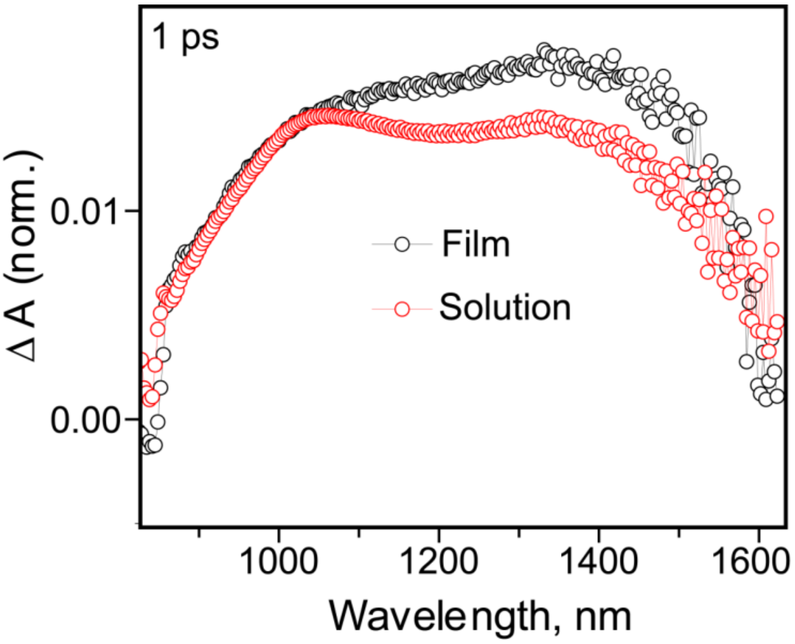

Supplementary Figure 28. Transient absorption spectra of TDPP-BBT copolymer in chlorobenzene and dried film with actinic pump at 650 nm. Transient spectral traces at 0.5 ps have been normalized for comparison.

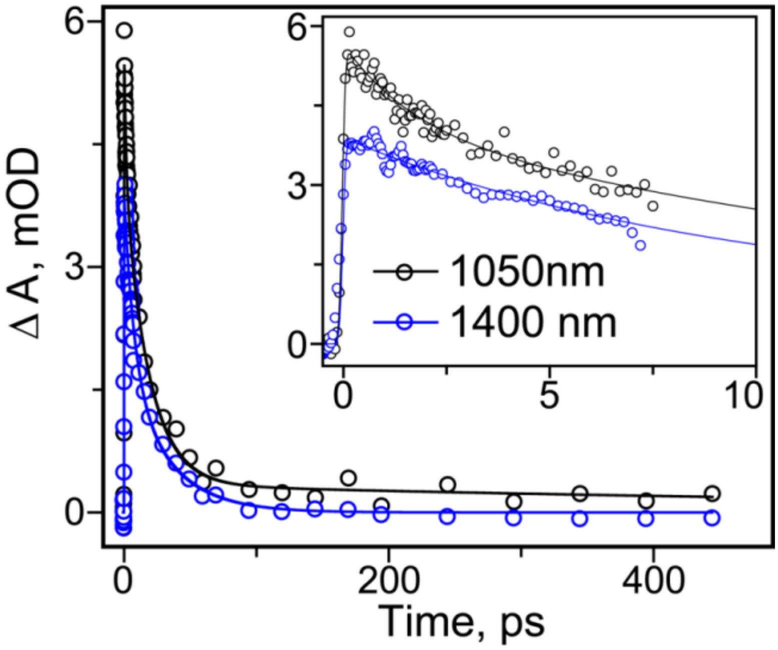

Supplementary Figure 29. TA of TDPP-BBT polymer film with 0.15 mW of 650 nm actinic pump. Kinetics fitting at 1050 nm and 1350 nm showing the presence of a short lived (near 35 ps) polaron pair and long lived (near 600 ps) exciton.

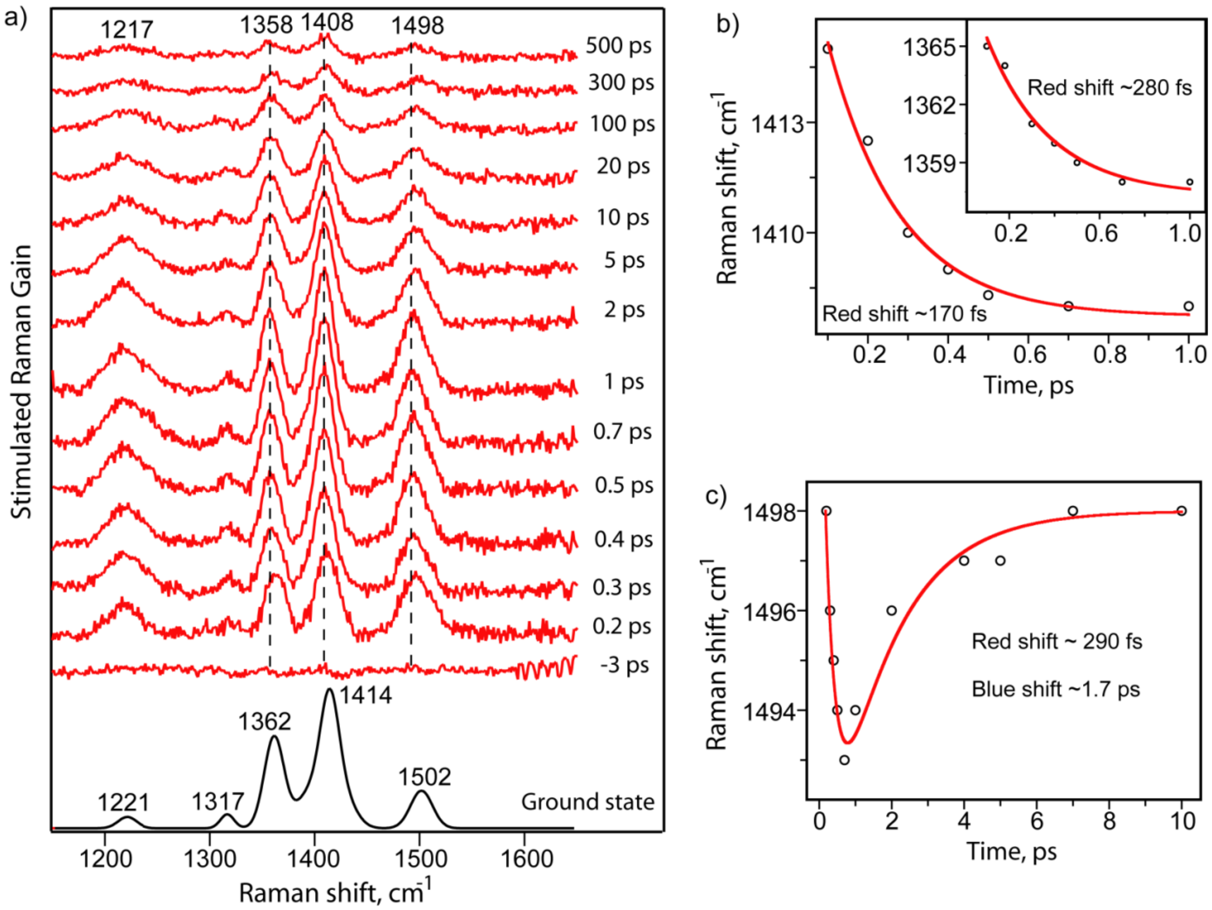

Supplementary Figure 30. a) Excited state Raman data of TDPP-BBT film at different time delays. b-c) The frequency shifts has been plotted. They show similar dynamics as observed for the TDPP-BBT solution in chlorobenzene.

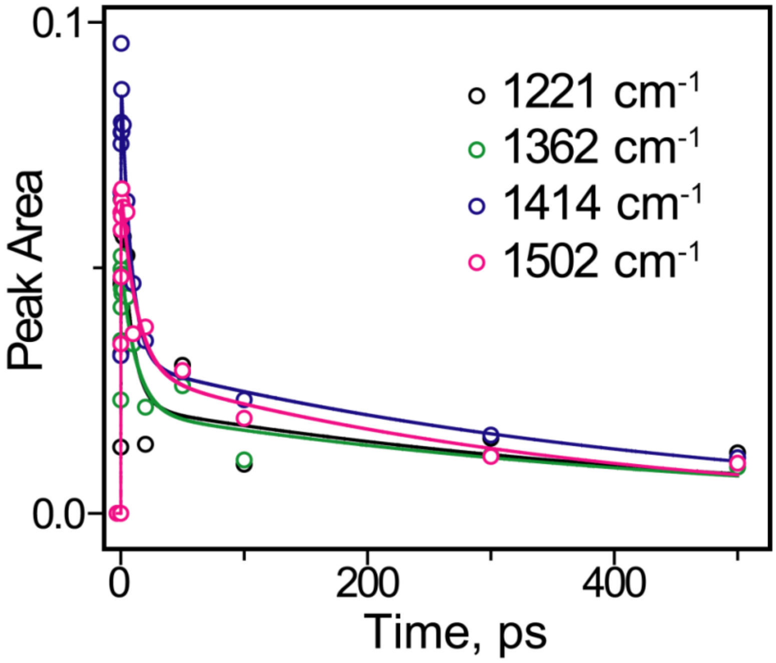

PeakArea	Rise (ps)	Decay1 (ps)	Decay2 (ps)
1221 cm^{-1} Peak	0.28	9	500
1362 cm^{-1} Peak	0.3	12	500
1414 cm^{-1} Peak	0.2	8	470
1502 cm^{-1} Peak	0.31	11	500

Supplementary Figure 31. Peak area kinetics for all the four modes as obtained from FSRS measurement for TDPP-BBT film as shown in Supplementary Figure 30.

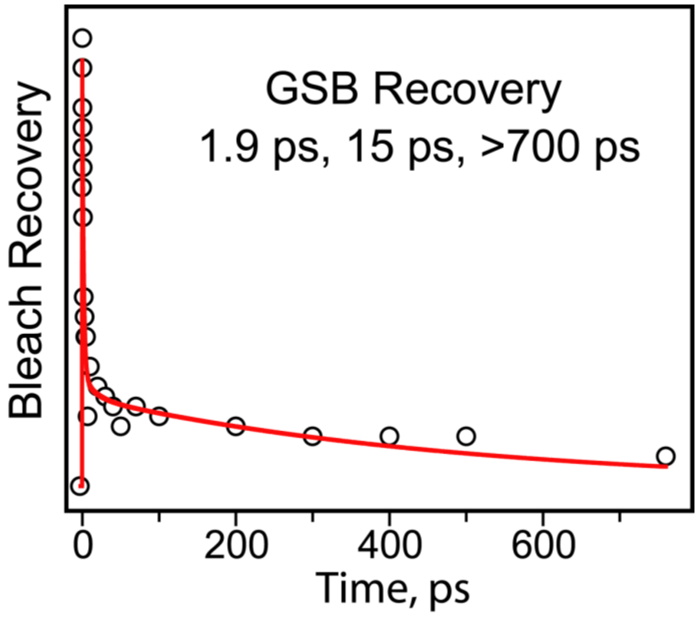

Supplementary Figure 32. Bleach recovery kinetics for FSRS data analysis for TDPP-BBT film showing a long lived component. This is similar to that obtained for TDPP-BBT in solution.

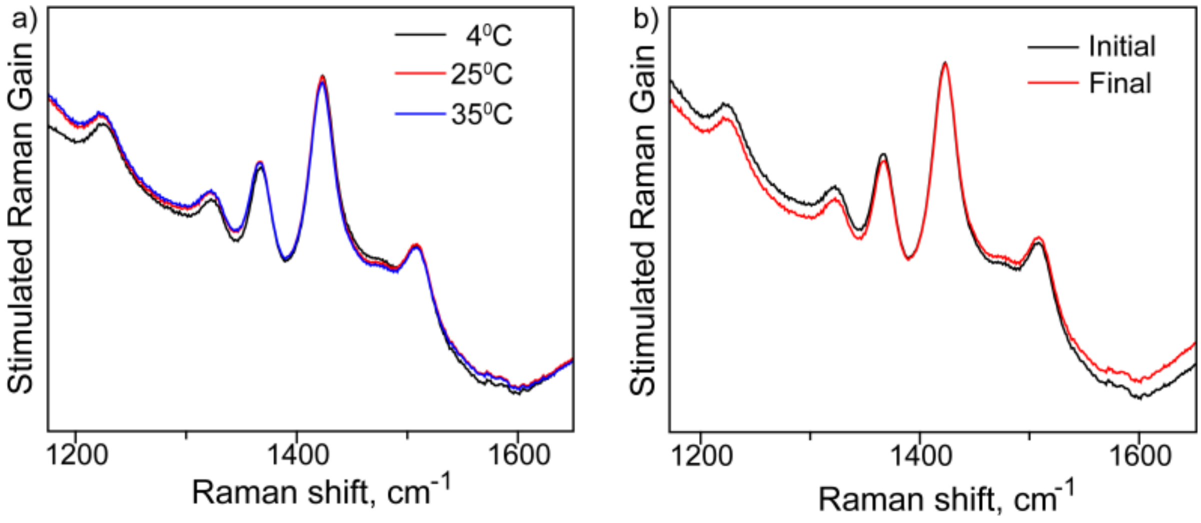

Supplementary Figure 33. Ground state stimulated Raman of the TDPP-BBT in chlorobenzene collected at a) different temperatures and b) before and after the FSRS measurement. Since we carried out rapid-flow measurements (Mathies et al. PNAS 1976) to avoid any photo-damage, we believe the effect of local heating can be avoided as the sample is kept in a reservoir at fixed temperature. Supplementary Figure 33b shows the integrity of the sample before and after measurements and find no change at all. Additionally we have performed temperature-dependent Raman measurements to check the effect of heating in Supplementary Figure 33a. We find the ground state Raman signal hardly changes in the temperature range from 4 degrees to 35 degrees centigrade.

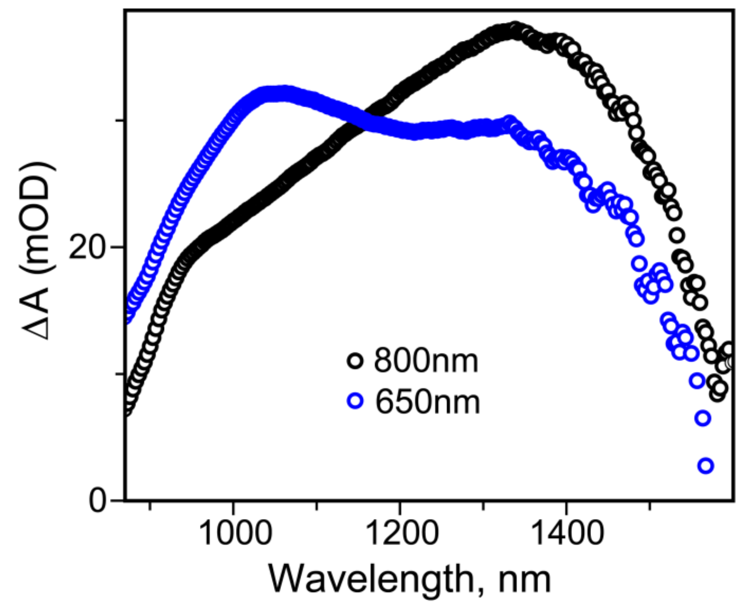

Supplementary Figure 34. TA spectra of TDPPBBT in CLB at 800 nm (black) and 650 nm (blue) pump pulses showing relative population near 1400 nm feature increases at 800 nm pump.

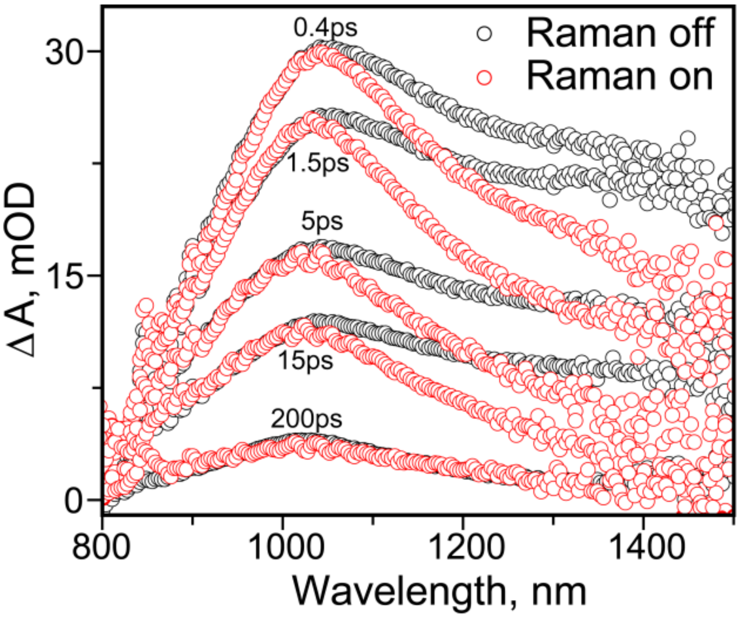

Supplementary Figure 35. TA spectra of TDPPBBT in CLB at 650 nm pump-in presence and absence of 1 mW of 816 nm Raman pump.

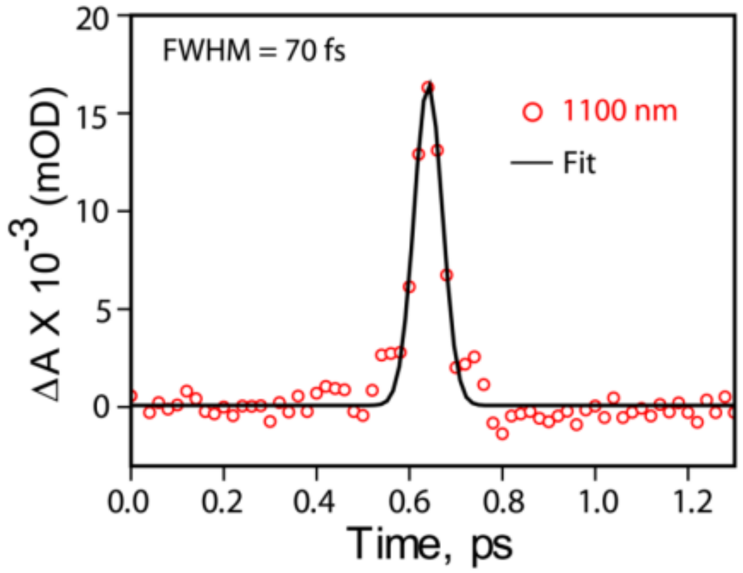

Supplementary Figure 36. Kerr signal with actinic pulse and probe pulse overlapped inside a 1 mm glass. The instrument response function for FSRS measurement as determined from the FWHM of the fit is 70 fs.

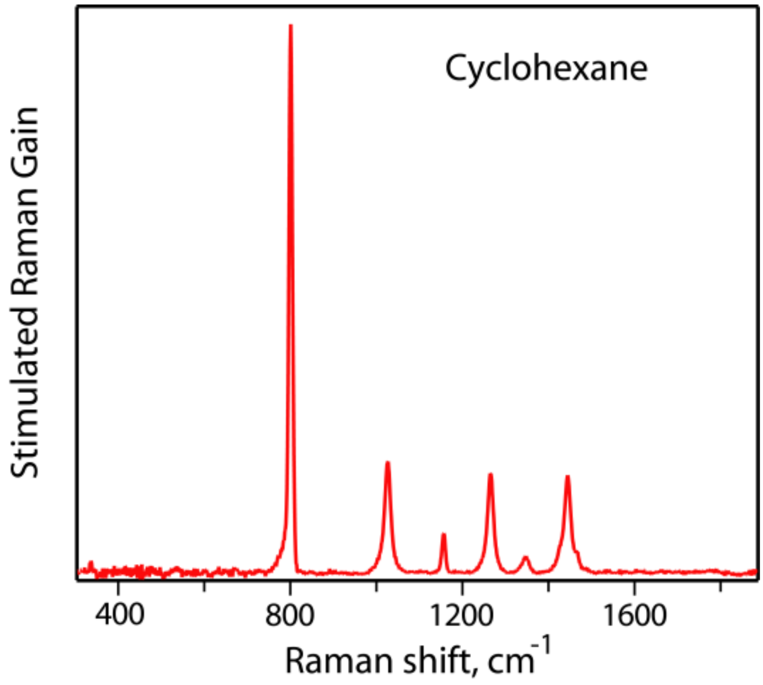

Supplementary Figure 37. Stimulated Raman spectra of cyclohexane in a 2 mm quartz cuvette. Raman pump wavelength used was 816 nm with 3 ps pulse width and 1 mW power. Spectral resolution as determined from the line width of the cyclohexane 802 cm^{-1} peak is 10 cm^{-1}.

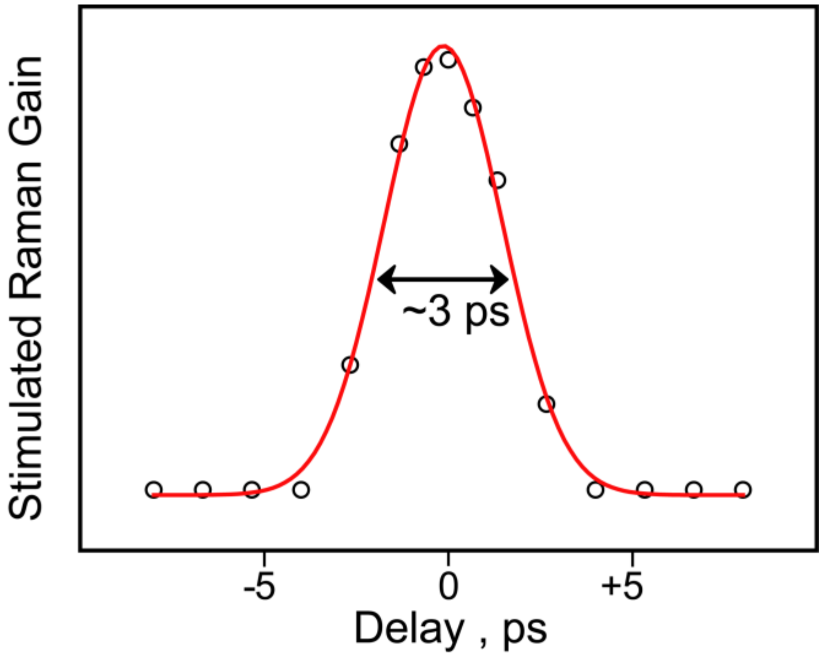

Supplementary Figure 38. Raman gain of cyclohexane 802 cm^{-1} peak is plotted as a function of Raman pump and probe delay. The FWHM of the fit provides the pulse width of 3 ps for Raman pump.

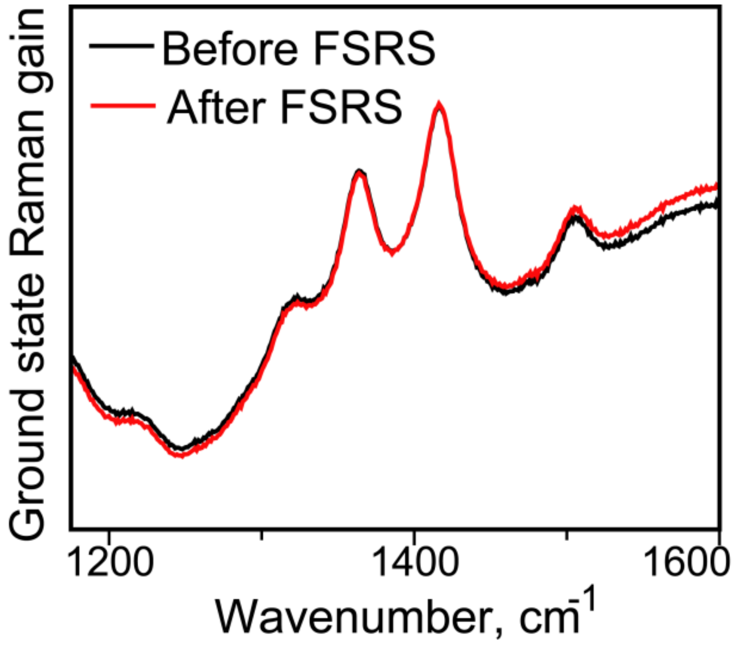

Supplementary Figure 39. The raw ground state stimulated Raman signal of TDPP-BBT film has been plotted before and after FSRS measurement to check for sample stability.

Supplementary Note 1: Synthesis of TDPP-BBT copolymer

Thiophene-diketopyrrolopyrrole–benzobithiophene (TDPP-BBT) based polymer (M_n = 9035 g mol^{-1}, regioregular) was synthesized as discussed in the following section. Chlorobenzene (CLB) (AR grade) was purchased from SD Fine-Chem Pvt. Ltd., India.

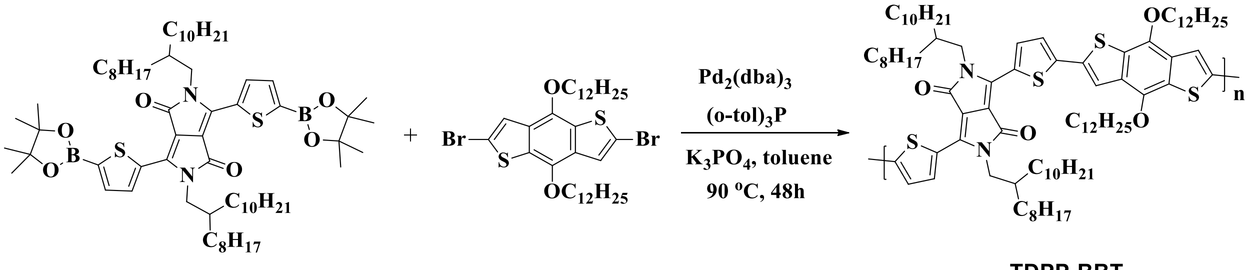

2,5-bis(2-octyldodecyl)-3,6-bis(5-(4,4,5,5-tetramethyl-1,3,2-dioxaborolan-2-yl)thiophen-2-yl pyrrolo[3,4-c]pyrrole-1,4(2H,5H)-dione **(M1)** (0.23 g, 0.2 mmol) and 2,6-dibromo-4,8-bis(dodecyloxy) benzo[1,2-b:4,5-b']dithiophene (0.15 g, 0.2 mmol) **(M2)** were taken in degassed dry toluene (20 mL) and two drop of aliquat336 were added. The reaction container was loaded with ligand (o-tol)$_3$P (15 mg), catalyst $Pd_2(dba)_3$ (10 mg) and purged with argon for 10 min to remove dissolved oxygen and was added aqueous (1 mL) solution of degassed K_3PO_4 (0.13g, 0.6 mmol). This mixture was refluxed at 90 °C for 48 h. Then cooled to room temperature and quenched with 1M HCl (10 mL) and then washed with EDTA solution, extracted with chloroform and dried over anhydrous Na_2SO_4, The organic layer was evaporated and concentrated solution was precipitated by methanol. Dark green precipitate was collected by filtration and then washed with hot methanol, acetone and hexane by soxhlet extraction to remove unreacted starting materials and lower molecular weight oligomers. The product was dried under vacuum to obtain pure TDPP-BBT as a dark green solid (0.2 g, 68%). M_n = 9.035 kDa, M_w = 28.42 kDa, PDI = 3.145

Supplementary Note 2: Sample preparation for measurements

4 ml of 2, 6 and 90 μM of pristine TDPP-BBT polymer solution in chlorobenzene was prepared for spectroscopic measurements. All these solutions were firstly argon purged and

then sealed. Subsequently these samples were heated for 2 hrs at 333 K and then brought at room temperature. For all steady-state and time-resolved measurements we used the above described sample preparation protocol. Solution of TDPP-BBT was chemically oxidized using ferric chloride as an oxidizing agent, and the absorption spectrum and stimulated Raman spectrum were recorded (see Supplementary Figure 15 and Supplementary Figure 21).

Supplementary Note 3: Kinetics data fitting procedure

The kinetic analyses of TCSPC, transient absorption and FSRS measurements were carried out using IGOR pro 5 wavemetrics software. This software has programs written to deconvolve the time-constants from the measured IRF. Fitting using this procedure provides decay time constants and their respective amplitudes values. The equation employed for fitting is given below:

$$\begin{aligned} y(t) = \quad & w_1\Bigg[\left(w_2 e^{\frac{[k_0^2-4bk_0(t-w_9)]}{4b}}\right)\mathrm{normdist}\left(\frac{2b(t-w_9)-k_0}{\sqrt{2b}}\right) \\ & + \left(w_4 e^{\frac{[k_1^2-4bk_1(t-w_9)]}{4b}}\right)\mathrm{normdist}\left(\frac{2b(t-w_9)-k_1}{\sqrt{2b}}\right) \\ & + \left(w_6 e^{\frac{[k_2^2-4bk_2(t-w_9)]}{4b}}\right)\mathrm{normdist}\left(\frac{2b(t-w_9)-k_2}{\sqrt{2b}}\right) \\ & + \left([-1-(w_4-w_6)]e^{\frac{[k_3^2-4bk_3(t-w_9)]}{4b}}\right)\mathrm{normdist}\left(\frac{2b(t-w_9)-k_3}{\sqrt{2b}}\right)\Bigg] \end{aligned} \quad \ldots [1]$$

Where, $b = \frac{4\ln 2}{w_0^2} k_0 = \frac{1}{w_3} k_1 = \frac{1}{w_5} \quad k_2 = \frac{1}{w_8}$

w_0 = instrument response function, IRF.

w_1 = scaling factor.

$w_2,\ w_4,\ w_6$ = amplitudes of the first, second, third exponentials.

$w_3,\ w_5,\ w_7,\ w_8\ =$ Four time constants.

$w_9\ =$ zero time.

Supplementary Note 4: FSRS signal processing

Stimulated Raman signal is obtained from the sample if both the Raman pump and probe pulses are overlapped temporally and spatially on the sample and we detect log ($I_{Raman+Probe}/I_{Probe}$). For FSRS experiments, we record two datasets: stimulated Raman spectra (SRS) without and with actinic pump. SRS without actinic pump contain ground state Raman spectra from both TDPP-BBT and solvent (see black spectrum in Supplementary Figure 1 and grey colour in Supplementary Figure 2). After solvent peak subtraction and baseline correction, we obtain ground state Raman spectra (GSR) (green spectrum in Supplementary Figure 1) of TDPP-BBT polymer. SRS with actinic pump (black spectrum in Supplementary Figure 2) can be processed to obtain excited Raman spectra (ESR) as described below.

The subtraction of SRS spectra (SRS with actinic pump on - SRS without actinic pump) give blue spectra in Supplementary Figure 2 that contain 1) bleach of ground state, 2) signal from excited state and 3) a baseline coming mainly from transient absorption and other nonlinear processes. To remove the ground state bleach contribution, we have added the ground state Raman spectra (GSR) with the fraction 'm' added till the bleach is completely removed and we get Gaussian shaped peak (orange spectrum). The values of '*m*' vary as the actinic pump delay time changes. Then the spectrum is baseline corrected to get only excited state Raman (ESR). This procedure is followed for several time delays and obtained FSRS spectra as shown in the main Figure 4. The values of '*m*' can be used to construct bleach recovery dynamics as shown in Supplementary Figure 19.

Supplementary Note 5: Metric for FSRS bleach filling and baseline correction

Supplementary Figure 3 shows the raw FSRS traces of pump-ON and pump-OFF Raman spectra. Along with it we have plotted the pure "ON"MINUS"OFF" data along with the added back GS spectrum that helps recover the pure excited state FSRS traces. Although the bleach dominates the "ON"MINUS"OFF" spectrum, the negative features are slightly blue-shifted (1422 cm^{-1} peak in the bleach appears at 1426 cm^{-1}) in the position as compared to pure ground state Raman peaks. The presence of blue-shifted bleach indicates the presence of an overlapping positive Raman feature on the red-side of the bleach which is obvious when we start adding back the ground state features. We define the coefficient "m" as the percentage ground state added back to recover the pure excited state Raman spectrum. In Supplementary Figure 3 the value of m is varied, and each of the added spectra are plotted for the time delay of 0.5 ps. The choice of "m" is constraint by both the removal of the negative features in the ground state added trace as well as the ground state recovery dynamics observed from the TA. We find that our metric "m" matches excellently with the ground state recovery dynamics from TA as compared in Supplementary Figure 4.

The raw Raman signal measured in our experiments contains a broad background in the "ON"MINUS"OFF" spectrum. The transient background in the "ON"MINUS"OFF" arises primarily from the transient absorption induced by the picosecond Raman pump along with a partial contribution from cross-phase modulation at early times near t=0. We record the TA data at all points with Raman-ON and use this to draw the baseline for the FSRS plots after ground state addition. It should be noted that we do not carry out any baseline correction in the individual raw Pump-ON or Pump-OFF data since the baseline in the individual spectra can arise from many non-linear contributions. In order to correct the baseline in the excited state FSRS spectrum after optimally adding ground state contribution, we draw smooth baselines which follow the major features observed in the transient absorption. A comparison

of the drawn baselines with the transient absorption traces is shown in Supplementary Figure 5-6.

Supplementary Note 6: Gaussian calculation and optimized Z-matrix

DFT calculation was done on TDPP-BBT repeating unit using B3LYP (6-31+G). Top and side view of the optimised structure is given in the Supplementary Figure 3. The corresponding Z- matrix table is also attached.

Optimized Structure Z-matrix

```
S
C  1  R12
C  1  R13      2  A213
C  3  R34      1  A134      2  -D2134
C  4  R45      3  A345      1  D1345
C  5  R56      4  A456      3  D3456
N  6  R67      5  A567      4  D4567
C  7  R78      6  A678      5  D5678
C  8  R89      7  A789      6  -D6789
C  9  R9_10      8  A89_10      7  D789_10
C  10  R10_11      9  A9_10_11      8  D89_10_11
C  11  R11_12      10  A10_11_12    9  D9_10_11_12
C  12  R12_13      11  A11_12_13    10  -D10_11_12_13
S  13  R13_14      12  A12_13_14    11  -D11_12_13_14
C  14  R14_15      13  A13_14_15    12  D12_13_14_15
C  15  R15_16      14  A14_15_16    13  -D13_14_15_16
C  16  R16_17      15  A15_16_17    14  -D14_15_16_17
C  17  R17_18      16  A16_17_18    15  -D15_16_17_18
C  18  R18_19      17  A17_18_19    16  D16_17_18_19
S  18  R18_20      17  A17_18_20    16  -D16_17_18_20
C  17  R17_21      18  A18_17_21    19  -D19_18_17_21
O  16  R16_22      15  A15_16_22    14  -D14_15_16_22
C  15  R15_23      14  A14_15_23    13  D13_14_15_23
C  23  R23_24      15  A15_23_24    16  D16_15_23_24
```

O 19 R19_25 18 A18_19_25 17 -D17_18_19_25

C 20 R20_26 18 A18_20_26 17 -D17_18_20_26

H 26 R26_27 20 A20_26_27 18 -D18_20_26_27

H 21 R21_28 17 A17_21_28 18 D18_17_21_28

H 24 R24_29 23 A23_24_29 15 -D15_23_24_29

S 12 R12_30 11 A11_12_30 10 D10_11_12_30

H 11 R11_31 12 A12_11_31 13 -D13_12_11_31

H 10 R10_32 11 A11_10_32 12 D12_11_10_32

C 8 R8_33 7 A78_33 6 D678_33

C 7 R7_34 6 A67_34 5 D567_34

O 6 R6_35 7 A76_35 8 -D876_35

C 33 R33_36 5 A5_33_36 6 D65_33_36

N 4 R4_37 5 A54_37 6 -D654_37

C 37 R37_38 36 A36_37_38 33 D33_36_37_38

O 36 R36_39 37 A37_36_39 4 -D4_37_36_39

H 38 R38_40 37 A37_38_40 4 D4_37_38_40

H 38 R38_41 37 A37_38_41 4 D4_37_38_41

H 38 R38_42 37 A37_38_42 4 -D4_37_38_42

H 34 R34_43 7 A7_34_43 8 D87_34_43

H 34 R34_44 7 A7_34_44 8 D87_34_44

H 34 R34_45 7 A7_34_45 8 -D87_34_45

C 25 R25_46 19 A19_25_46 18 -D18_19_25_46

C 22 R22_47 16 A16_22_47 17 -D17_16_22_47

H 46 R46_48 25 A25_46_48 19 D19_25_46_48

H 46 R46_49 25 A25_46_49 19 D19_25_46_49

H 46 R46_50 25 A25_46_50 19 -D19_25_46_50

H 47 R47_51 22 A22_47_51 16 -D16_22_47_51

H 47 R47_52 22 A22_47_52 16 D16_22_47_52

H 47 R47_53 22 A22_47_53 16 -D16_22_47_53

C 3 R3_54 1 A13_54 2 -D213_54

C 54 R54_55 3 A3_54_55 4 D43_54_55

H 54 R54_56 55 A55_54_56 2 D2_55_54_56

H 55 R55_57 2 A2_55_57 1 D12_55_57

H 2 R2_58 1 A12_58 3 -D312_58

R12 = 1.72305212	R17_21 = 1.44065054
R13 = 1.76097197	A18_17_21 = 111.71649587
A213 = 91.57174924	D19_18_17_21 = 178.76848780
R34 = 1.44345679	R16_22 = 1.37809340
A134 = 120.34453968	A15_16_22 = 120.58548749
D2134 = 179.53214721	D14_15_16_22 = 1.37028584
R45 = 1.39230952	R15_23 = 1.42676162
A345 = 129.22139297	A14_15_23 = 111.39256347
D1345 = 16.56002885	D13_14_15_23 = 1.48267792
R56 = 1.44766187	R23_24 = 1.43285682
A456 = 142.04551129	A15_23_24 = 111.91686409
D3456 = 3.15993047	D16_15_23_24 = 178.54607745
R67 = 1.43522648	R19_25 = 1.37572303
A567 = 103.99372087	A18_19_25 = 120.56885343
D4567 = 173.63321325	D17_18_19_25 = 178.23302014
R78 = 1.39953428	R20_26 = 1.75238726
A678 = 111.48014549	A18_20_26 = 90.56031429
D5678 = 0.42553920	D17_18_20_26 = 0.87251659
R89 = 1.43893983	R26_27 = 1.08298851
A789 = 124.28898798	A20_26_27 = 119.00383045
D6789 = 178.79842007	D18_20_26_27 = 179.42855537
R9_10 = 1.39369470	R21_28 = 1.08397917
A89_10 = 130.11727285	A17_21_28 = 123.52406907
D789_10 = 11.58509551	D18_17_21_28 = 178.52001828
R10_11 = 1.40778634	R24_29 = 1.08456183
A9_10_11 = 113.86046519	A23_24_29 = 123.08068234
D89_10_11 = 179.13113256	D15_23_24_29 = 176.97248742
R11_12 = 1.38721453	R12_30 = 1.74454700
A10_11_12 = 113.40016127	A11_12_30 = 110.93700928
D9_10_11_12 = 0.02018630	D10_11_12_30 = 0.04734696
R12_13 = 1.44640034	R11_31 = 1.08498751
A11_12_13 = 128.22746724	A12_11_31 = 123.19536202
D10_11_12_13 = 179.53578541	D13_12_11_31 = 0.98637247
R13_14 = 1.77621840	R10_32 = 1.08044006
A12_13_14 = 119.76005636	A11_10_32 = 122.29489709
D11_12_13_14 = 16.31802411	D12_11_10_32 = 178.15707682
R14_15 = 1.75683203	R8_33 = 1.39465804
A13_14_15 = 90.90890799	A78_33 = 106.71900064
D12_13_14_15 = 179.14794528	D678_33 = 0.39227566
R15_16 = 1.39116209	R7_34 = 1.45254143
A14_15_16 = 126.08160005	A67_34 = 119.29481257
D13_14_15_16 = 178.64497761	D567_34 = 174.42895812
R16_17 = 1.40714635	R6_35 = 1.22755113
A15_16_17 = 118.11418456	A76_35 = 123.01018856
D14_15_16_17 = 178.96342974	D876_35 = 178.20959101
R17_18 = 1.42615423	R33_36 = 1.44722426
A16_17_18 = 119.39816842	A5_33_36 = 108.23761225
D15_16_17_18 = 0.98045193	D65_33_36 = 178.05130904
R18_19 = 1.39336858	R4_37 = 1.39779319
A17_18_19 = 122.50719806	A54_37 = 106.81227231
D16_17_18_19 = 0.93962488	D654_37 = 175.83047135
R18_20 = 1.75827835	R37_38 = 1.45298225
A17_18_20 = 111.29194102	A36_37_38 = 119.46355770
D16_17_18_20 = 179.29831655	D33_36_37_38 = 173.74575971
A37_36_39 = 122.92470276	R36_39 = 1.22863175
D4_37_36_39 = 178.07052486	A25_46_49 = 110.77056656
R38_40 = 1.09198346	D19_25_46_49 = 60.68560089
A37_38_40 = 106.62209976	R46_50 = 1.09663645
D4_37_38_40 = 162.02279893	A25_46_50 = 111.06650842
R38_41 = 1.09367359	D19_25_46_50 = 61.23700147
A37_38_41 = 110.82318233	R47_51 = 1.09203707

D4_37_38_41 = 42.73177150<br>R38_42 = 1.09604928<br>A37_38_42 = 111.80923205<br>D4_37_38_42 = 79.90988637<br>R34_43 = 1.09201254<br>A7_34_43 = 106.45533131<br>D87_34_43 = 165.83178010<br>R34_44 = 1.09419430<br>A7_34_44 = 111.02011830<br>D87_34_44 = 46.76322241<br>R34_45 = 1.09592093<br>A7_34_45 = 111.78143619<br>D87_34_45 = 76.11238538<br>R25_46 = 1.43665803<br>A19_25_46 = 115.09529930<br>D18_19_25_46 = 97.48637624<br>R22_47 = 1.43560547<br>A16_22_47 = 114.77920017<br>D17_16_22_47 = 86.81025811<br>R46_48 = 1.09175606<br>A25_46_48 = 105.99006009<br>D19_25_46_48 = 179.69519038<br>R46_49 = 1.09692497	A22_47_51 = 106.06359296<br>D16_22_47_51 = 179.55423233<br>R47_52 = 1.09704304<br>A22_47_52 = 111.02040452<br>D16_22_47_52 = 61.29813314<br>R47_53 = 1.09700299<br>A22_47_53 = 110.88478734<br>D16_22_47_53 = 60.53154705<br>R3_54 = 1.39120495<br>A13_54 = 109.93636843<br>D213_54 = 0.77008254<br>R54_55 = 1.41651291<br>A3_54_55 = 113.56873514<br>D43_54_55 = 179.06815056<br>R54_56 = 1.08111824<br>A55_54_56 = 122.70477685<br>D2_55_54_56 = 178.45203077<br>R55_57 = 1.08470041<br>A2_55_57 = 123.77342187<br>D12_55_57 = 178.85378948<br>R2_58 = 1.08280866<br>A12_58 = 119.69463937<br>D312_58 = 179.81115922

The experimentally determined Raman peaks for TDPP-BBT copolymer has been assigned based on the DFT calculation on TDPP-BBT repeating unit. The frequencies of 125, 1228, 1320, 1367, 1422 and 1509 cm^{-1} have been depicted in Supplementary Figure 9.
